# Disentangling individual differences in cognitive response mechanisms for rating scale items: A flexible-mixture multidimensional IRTree approach

**DOI:** 10.3758/s13428-025-02778-0

**Published:** 2025-08-13

**Authors:** Ömer Emre Can Alagöz, Thorsten Meiser, Lale Khorramdel

**Affiliations:** 1 Department of Psychology, University of Mannheim, L 13 15, 68161 Mannheim, Germany; 2 National Board of Medical Examiners (NBME), Philadelphia, USA

**Keywords:** Response processes, Heterogeneity, Response styles, Item response theory, Mixture modeling

## Abstract

The accuracy of our inferences from rating-scale items can be improved with IRTree models, which consider heuristic response strategies like response styles (RS). IRTree models break down ordinal responses into pseudo-items (nodes), each representing a distinct decision-making process. These nodes are then modeled using an item response model. In the case of four-point items, a response is split into two nodes: 1) response direction, where the trait influences the overall agreement with items, and 2) response extremity, where both the trait and extreme RS (ERS) impact the choice of relative (dis)agreement categories. However, traditional models, despite addressing RS effects, assume that all respondents follow an identical response strategy, where the selection of relative (dis)agreement categories is influenced by the trait and ERS to the same degree for all respondents. Given that respondents may vary in the extent to which they adopt heuristic-driven strategies (e.g., fatigue, motivation, expertise), this assumption of homogeneous response processes is unlikely to be satisfied, potentially leading to inaccurate inferences. To accommodate different response strategies, we introduce the mixture IRTree model (MixTree). In MixTree, participants are assigned to different latent classes, each associated with distinct response processes. Based on their class memberships, varying weights are assigned to individuals’ trait and ERS scores. Additionally, MixTree simultaneously examines extraneous variables to explore sources of heterogeneity. A simulation study validates the MixTree’s performance in recovering classes and model parameters. Empirical data analysis identifies two latent classes, one linked to a trait-driven and the other to RS-driven mechanisms.

## Introduction

Questionnaires with rating-scale items are frequently used in psychological and educational research to measure unobservable constructs as they provide first-hand information in a cost- and time-efficient way. Although they provide researchers with efficiency, it is expected that respondents need to go through a four-step cognitive process to provide a response to each item (Tourangeau et al., 2000). These four steps include comprehension of the item content, searching and retrieving the relevant information from memory, integration and synthesis of the retrieved information into a judgement, and finally, mapping this judgement to the response option that is perceived as the most suitable.

A successful completion of all four steps is referred to as *optimizing*, a strategy where respondents spend adequate cognitive effort to provide the most accurate response according to their substantive trait level (Krosnick, 1991; Krosnick & Alwin, 1987). However, some respondents may complete some of the steps only superficially or even skip them to preserve their cognitive resources, which is referred to as *satisficing* strategy (Krosnick, 1991; Krosnick & Alwin, 1987). Satisficing can develop over the course of a questionnaire (Merhof & Meiser, 2023) with the fatigue effect, but can also be adopted at the beginning of a questionnaire when, for instance, respondents are unmotivated, not rewarded for their participation, or were frequently administered questionnaires before (e.g., university students due to convenience sampling).

Satisficing and optimizing strategies are sometimes perceived as mutually exclusive categories, with respondents either fully engaging with the cognitive response process or opt for minimal participation. In contrast, these strategies were argued to exist on a continuum (Krosnick, 1991; Roberts et al., 2019; Tourangeau, 2018), suggesting that engagement of respondents differ gradually. Furthermore, several individual factors were suggested to affect where on this satisficing–optimizing continuum a respondent stands, such as cognitive abilities, motivation (Krosnick, 1991, 1999), experience with the trait being measured (Tourangeau, 2018), the need for cognition (Cacioppo & Petty, 1982), or personality traits (Sturgis & Brunton-Smith, 2023).

Respondents with high levels of satisficing employ alternative response strategies to alleviate the cognitive burden of responding, including the use of response styles (RS; Roberts et al., 2019). RS refer to systematic tendencies towards choosing specific response categories, irrespective of the item content (Baumgartner & Steenkamp, 2001; Paulhus, 1991; Podsakoff et al., 2003). For instance, extreme RS (ERS) is the tendency towards choosing extreme categories regardless of the actual content of the question. Indeed, using RS can significantly reduce the cognitive effort, as it is a systematic and consistent strategy (both within and between questionnaires; Wetzel et al., 2013; 2016), requiring no processing of content-relevant information from items.

Respondents may adopt RS when they allocate less (or no) effort to any of the four steps in the response process (Roberts, 2016). If the comprehension step is given less weight, respondents may anchor their response to the general theme of the questionnaire and avoid fine-tuning their category choices for their substantive trait levels. Similarly, a reduced effort in the retrieval step may result in limited information about the item-specific aspect of the trait (Weber & Johnson, 2006), which opens up space for heuristic strategies like RS and results in an inability to make detailed judgements. Even if the retrieval step was executed, factors such as fatigue or lack of motivation may interfere with their integration and synthesis at the third step. Finally, regardless of whether the previous steps were successfully executed, unfamiliarity with rating scale items, high number of response options, and inability to make sense of category labels may cause reduced effort or failure in distinguishing response categories (Blasius & Thiessen, 2012; Krosnick & Alwin, 1987; Krosnick et al., 2002). Hence, an observed response may reflect more than the underlying trait level if the cognitive processes involve heuristic strategies, and modeling such strategies is crucial for the validity of statistical inferences.

## Modeling individual differences in the use of RS

Modeling RS effects has always been of interest as they distort the true category choices of respondents (Van Vaerenbergh & Thomas, 2013). When not controlled for, RS bias the trait (or sum) scores, item estimates (Bolt & Johnson, 2009), factor structures (D’Urso et al., 2023), correlations between traits (Böckenholt & Meiser, 2017; von Davier & Khorramdel, 2013; Khorramdel & von Davier, 2014), and comparative tests between groups of interest (Cheung & Rensvold, 2000; Ulitzsch et al., 2024). In such cases, researchers face a crucial threat to the validity of their statistical inferences about the measured trait.

There are various psychometric models for modeling RS effects from different frameworks, such as confirmatory factor analysis or latent class analysis models, but here we focus on Item Response Theory (IRT) models (see Henninger & Meiser, 2020a; b, for an overview of different models). Within the IRT framework, the item response tree (IRTree) model family is widely used for accounting RS effects (Böckenholt, 2012; De Boeck & Partchev, 2012; Jeon & De Boeck, 2016). IRTree models consider a response as a product of several decision-making processes and associate different decisions with different factors, usually the response direction with the trait and relative category choices with both the trait and RS factors (Böckenholt & Meiser, 2017; Khorramdel & von Davier, 2014; Meiser et al., 2019). Individual differences in content-irrelevant response tendencies are then captured with RS factor scores, such as a higher ERS score increases the probability of choosing an extreme category over non-extreme categories.

Traditional IRTree models, however, have a major disadvantage. Although they account for individual differences in category tendencies, they overlook the differences in response strategies. That is, individuals may put different relative weights on the trait and RS factors while executing the cognitive steps due to the reasons explained before. However, IRTree models assume that the weights given to the trait and RS factors are homogeneous in the entire population, implying an identical response strategy across all respondents. In other words, IRTree models assume that all respondents make relative category choices by utilizing the trait and RS factors to the same extent.

There are several models proposed for capturing differences in response strategies. Tijmstra et al. (2018) proposed a mixture item response model to capture two types of respondents, those who make trait-based responses for all categories and those who additionally employ a midscale RS (MRS; tendency towards the middle category). Kim and Bolt (2021) proposed a mixture IRTree model to disentangle respondents, who give purely trait-based responses and those who partially use ERS in their response strategy. Recently, Alagöz and Meiser (2024) proposed a mixture IRTree model that differentiates four types of response strategies, namely a strategy based consistently on the trait factor (no RS), and other strategies that additionally use ERS, MRS, or both ERS and MRS.

All these models indeed detected noticeable proportions of different strategies in empirical data. However, they all differentiate respondents based on whether they use the relevant RS or not in a binary fashion. From the satisficing framework described before, they assume that there are satisficers and optimizers in the sample, and that satisficing and optimizing are binary outcomes. Because the extent of satisficing can differ between respondents, the reality might be more complex than binary classifications of respondents into one of the two strategies.

To remedy the binary view in previous research, here we propose a mixture IRTree (MixTree) model that captures latent subpopulations of respondents who gradually differ in their response strategies. More specifically, the MixTree model allows for latent subpopulations where the weights assigned to the trait and RS in the cognitive response process can differ. In other words, MixTree can capture respondents who show different levels of satisficing. Furthermore, the MixTree model further allows researchers to model the predictors of class memberships (such as demographics or process data), which can unravel sources of heterogeneity.

The proposed MixTree model also serves as a general framework that captures the previous mixture models proposed by Alagöz and Meiser (2024) and Kim and Bolt (2021) as its special cases. That is, by fixing the weights of the trait or relevant RS, these previous models can be expressed with the MixTree model. Furthermore, the MixTree model offers various extensions that expand to modeling of other heuristics, such as acquiescence RS or effortless responding, which are discussed in the *Discussion* section.

In the next section, we describe the new MixTree model, then we present a simulation study to assess model performance regarding classifications and parameter recovery, and, finally, we illustrate the model with an empirical example.

### IRTree models

IRTree models consider a response as a product of several decision-making processes (i.e., nodes). Each decision usually has a binary outcome, and the rating responses are then decomposed into pseudo-items representing the outcomes of decisions. These pseudo-items can be analyzed with an item response model (e.g., 2-PL) to disentangle factors affecting each decision.

**Fig. 1:**
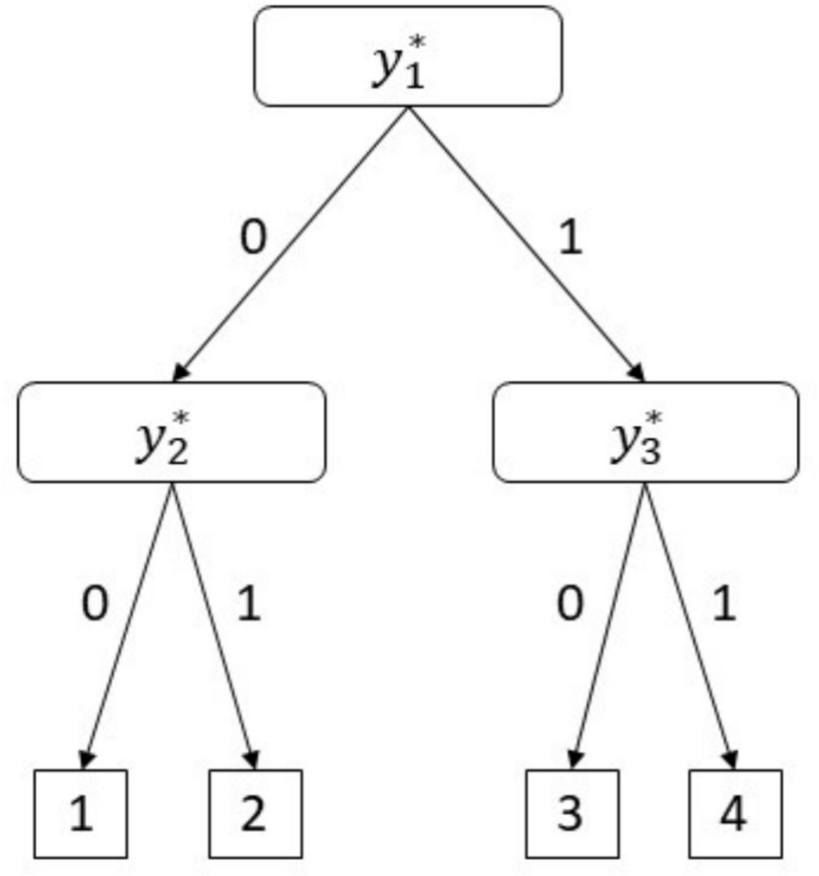
IRTree structure for four-point rating scale items

#### The Non-Mixture IRTree Model

IRTree models are often used in separating the trait from RS effects. In the case of four-point rating scale items, as illustrated in Fig. 1, respondents make two decisions until they respond to an item^1^. The first decision (${y_{1}^{*}}$) is about the response direction. Respondents either take the disagreement (${y_{1}^{*}}=0$) or the agreement (${y_{1}^{*}}=1$) direction. Given the response direction, respondents decide whether they strongly (dis)agree (${y_{2}^{*}}=0$ or ${y_{3}^{*}}=1$) or just (dis)agree (${y_{2}^{*}}=1$ or ${y_{3}^{*}}=0$). The full set of pseudo-item coding for each original response is provided in Table 1.

**Table 1:** Pseudo-item decomposition for four-point rating scale items

Pseudo-items	Ordinal response				Traditional IRTree	MixTree
	1	2	3	4	$P({y}_{tpj}^{*}=1)$	$P({y}_{tpj}^{*}=1|{X=c})$
$y_{1pj}^*$	0	0	1	1	$g^{-1}(\alpha _{j}^{(trait)}\theta _{p} + \beta _{1j})$	$g^{-1}(\alpha _{j}^{(trait)}\theta _{p} + \beta _{1j})$
$y_{2pj}^*$	0	1	–	–	$g^{-1}(\alpha _{j}^{(trait)}\omega _{j}\theta _{p} - \alpha ^{(ers)}\eta _{p} + \beta _{2j})$	$g^{-1}(\alpha _{j}^{(trait)}\omega _{jc}\theta _{p} - \alpha ^{(ers)}_{c}\eta _{p} + \beta _{2jc})$
$y_{3pj}^*$	–	–	0	1	$g^{-1}(\alpha _{j}^{(trait)}\omega _{j}\theta _{p} + \alpha ^{(ers)}\eta _{p} + \beta _{3j})$	$g^{-1}(\alpha _{j}^{(trait)}\omega _{jc}\theta _{p} + \alpha ^{(ers)}_{c}\eta _{p} + \beta _{3jc})$

*Note.* ’-’ denotes missing-by-design, as a respondent who agrees (disagrees) with the item does not make the extreme decision on the disagreement (agreement) side

To model the direction decision, one can employ a unidimensional 2-PL model, where the substantive trait determines the response direction. To model the extremity decisions, one can employ multidimensional 2-PL models, where the decision is made based on both the substantive trait and ERS factor (Meiser et al., 2019). Specifically, let ${p} \in \{1,...,p,..,N\}$ denote a respondent, ${j}\in \{1,...,j,..,J\}$ denote an item, and ${g}^{-1}$ denote the inverse logit function. Then the probability of observing a response vector $\mathbf {{Y}_{p}}$ of length *J* is the product of the item-specific decision probabilities across *J* items:

1 $$\begin{aligned} P(\mathbf {Y_p})= &  \prod _{j=1}^{J} P(y^*_{1pj}) \times P(y^*_{2pj})^{{(1-y^{*}_{1pj})}} \times P(y^*_{3pj})^{(y^{*}_{1pj})} \nonumber \\= &  \prod _{j=1}^{J} \left( g^{-1}\left( y^*_{1pj}\left[ \alpha _{j}^{(trait)}\theta _{p}+\beta _{1j}\right] \right) \times \right. \nonumber \\ &  \quad \left. g^{-1}\left( y^*_{2pj}\left[ \alpha _{j}^{(trait)}\omega _{j}\theta _{p}-\alpha ^{(ers)}\eta _{p}+\beta _{2j}\right] \right) ^{(1-y^{*}_{1pj})} \times \right. \nonumber \\ &  \quad \left. g^{-1}\left( y^*_{3pj}\left[ \alpha _{j}^{(trait)}\omega _{j}\theta _{p}+\alpha ^{(ers)}\eta _{p}+\beta _{3j}\right] \right) ^{(y^{*}_{1pj})}\right) \end{aligned}$$

In Eq. 1, $\alpha _{j}^{(trait)}$ is the factor loading of the trait $\theta$, and $\beta _{1j}$ is the intercept term at the response direction node. At this node, a higher trait score $\theta$ increases the probability of agreeing with the item content. In the extreme decision nodes ${y_{2}^{*}}$ and ${y_{3}^{*}}$, $\omega _{j}$ are the proportionality constants for the factor loading of the trait relative to the direction node (Alagöz & Meiser, 2024; Quirk & Kern, 2023). Therefore, the $\omega _{j}$ constant implies that the trait plays a role in the extreme category choices to an extent that is proportional to its effect at the response direction node. Therefore, $\omega _{j}<1$ ($\omega _{j}>1$) implies that the effect of trait is weaker (stronger) for specific category decisions than for the response direction decision. Then $\alpha ^{(ers)}$ denotes the factor loading of the ERS factor $\eta$ at both extreme decision nodes. For a constant $\eta$, higher $\theta$ scores increase the probability of higher categories ("2" instead of "1" for the disagreement direction and "4" instead "3" for the agreement direction). For a constant $\theta$, higher $\eta$ scores increase the probability of extreme categories ("1" instead of "2" and "4" instead of "3" given the response direction). Finally, $\beta _{2j}$ and $\beta _{3j}$ are the intercept terms at the extreme decision nodes. Note that the ERS factor loading $\alpha ^{(ers)}$ is specified as item-invariant as the definition of RS suggests that such tendencies are independent of the item content. However, item features, such as length, complexity, or wording, may cause less engagement for a specific item. In order to comply with this theoretical basis, the proportionality constant is specified item-specific to allow for varying relative strengths of the trait and RS in the item-specific nodes.

As is clear from Eq. 1, the common IRTree approach strictly assumes that a single response strategy is adopted by all respondents. Assuming equal variances for the trait and ERS factors ($\sigma ^{2}_{\theta }=\sigma ^{2}_{\eta }=1$; as assumed by many for metric identification), $\alpha _{j}^{(trait)}\omega _{j}$ and $\alpha ^{(ers)}$ quantify the relative impact of the trait and ERS factor on the category choices, respectively, and are identical for *all* respondents. Given there is heterogeneity in response strategies, such as between-person differences in the relative weights associated with the trait and ERS, the traditional IRTree approach would fail to account for it.

#### The Mixture IRTree (MixTree) Model

In case of heterogeneous response strategies, a mixture model can be used for accommodating different subpopulations and for estimating class-specific parameters. Below, we describe the MixTree model. Let *X* be a discrete latent variable with realization $c \in \{1,...,c,..., C\}$. Then the probability of observing a response vector $\mathbf {{Y}_{p}}$ of length *J* given a covariate vector $\mathbf {{Z}_{p}}$ of length *K* is the weighted sum of the product of class- and item-specific decision probabilities across *J* items:

2 $$\begin{aligned} \begin{aligned} P(\mathbf {Y_p} | \mathbf {Z_p})&= \sum _{c=1}^{C} P(X=c | \mathbf{{Z_p}}) \prod _{j=1}^{J} P(y^*_{1pj} | X=c) \times \\&\quad P(y^*_{2p} | X=c)^{{(1-y^{*}_{1pj})}} \times P(y^*_{3p} | X=c)^{(y^{*}_{1pj})} \\&= \sum _{c=1}^{C} \pi _{pc} \prod _{j=1}^{J} \biggl ( g^{-1}\left( y^*_{1pj}\left[ \alpha ^{(trait)}_{j}\theta _{p}+\beta _{1j}\right] \right) \times \\&\quad g^{-1}\left( y^*_{2pj}\left[ \alpha ^{(trait)}_{j}\omega _{jc}\theta _{p}-\alpha ^{(ers)}_{c}\eta _{p}+\beta _{2jc}\right] \right) ^{(1-y^*_{1pj})} \times \\&\quad g^{-1}\left( y^*_{3pj}\left[ \alpha ^{(trait)}_{j}\omega _{jc}\theta _{p}+\alpha ^{(ers)}_{c}\eta _{p}+\beta _{3jc}\right] \right) ^{(y^*_{1pj})}\biggr ) \end{aligned} \end{aligned}$$

The response direction node ($y^{*}_{1}$) is specified class-invariant, implying that all respondents show at least some engagement with the item to the same extent to assess their stance on a binary level (disagree vs. agree). This assumption is in line with the satisficing framework that respondents do not respond randomly but minimize their effort to provide a good-enough response (see *Discussion* for potential model extension to account for non-effortful responding in case of a full disengagement from the response process). Furthermore, this invariance assumption also ensures that the MixTree model captures heterogeneity only in how specific categories are selected rather than changes in the nature of the substantive trait.

At the extremity decision nodes ($y^{*}_{2}$ and $y^{*}_{3}$), we let the proportionality constant $\omega _{jc}$ be class-specific, which then allows respondents to base their decisions on the trait to varying degrees. Similarly, the ERS factor loading $\alpha ^{(ers)}_{c}$ is also made class-specific, implying that respondents can make use of their heuristic strategies to varying degrees. Finally, node intercepts $\beta _{2jc}$ and $\beta _{3jc}$ are also made class-specific to capture between-class differences in the overall tendency to avoid extreme disagreement and choose extreme agreement categories.

Furthermore, the term $P(X=c|\textbf{Z}_{p})$ implies that the class probabilities of respondents can be predicted by the covariates, allowing us to understand potential sources of the found heterogeneity. Such effects (null or substantial), can be captured by means of multinomial logistic regressions. Further details are provided in the “Estimation” section, where we describe the three-step maximum likelihood (ML) approach to estimate the MixTree model.

Given the scales of the latent variables are fixed via setting class-invariant expectations and a variance-covariance matrix, as explained later in the “Estimation” section, the class-specific parameters offer a valuable comparison within- and between-classes regarding the relative impact of the trait and ERS factors on specific decision processes. Since the latent variances of $\theta$ and $\eta$ are equally fixed at "1", the loading parameters $(\alpha ^{(trait)}_{j}\omega _{jc})$ and $(\alpha ^{(ers)}_{c})$ related to the proportion of variance that can be attributed to each factor. Therefore, within a class, the loadings of the trait $(\alpha ^{(trait)}_{j}\omega _{jc})$ and ERS $(\alpha ^{(ers)}_{c})$ can be compared to see whether and which factor explains larger variance in the decision outcomes compared to the other factor. Between classes, the trait factor loadings can be directly compared as the trait is linked between classes with the invariant first node across classes, but the ERS factor is not linked in such a way and a direct comparison would be invalid. However, the inferences for the within-class differences can be compared across classes to understand if the relative impact of the trait and ERS differs between classes. That is, one can compare if the relative impact of the trait and ERS on response decisions is different between classes. For example, if one observe $(\alpha ^{(trait)}_{j}\omega _{jc})$ is greater than $(\alpha ^{(ers)}_{c})$ in one class and the other way around in the other class, the conclusion would be that the former class is associated with trait-dominated and the latter class is associated with a heuristic-dominated response strategy. Therefore, relative weights of the trait and ERS that are captured by factor loadings can help us interpret whether either of both play a greater role in fine-grained category choices given the response direction in different classes. A hypothetical example given in the next section and the empirical illustration in Section “Empirical illustration: Baron-Cohen’s systemizing quotient test” illustrate this feature of the MixTree model.

**Fig. 2:**
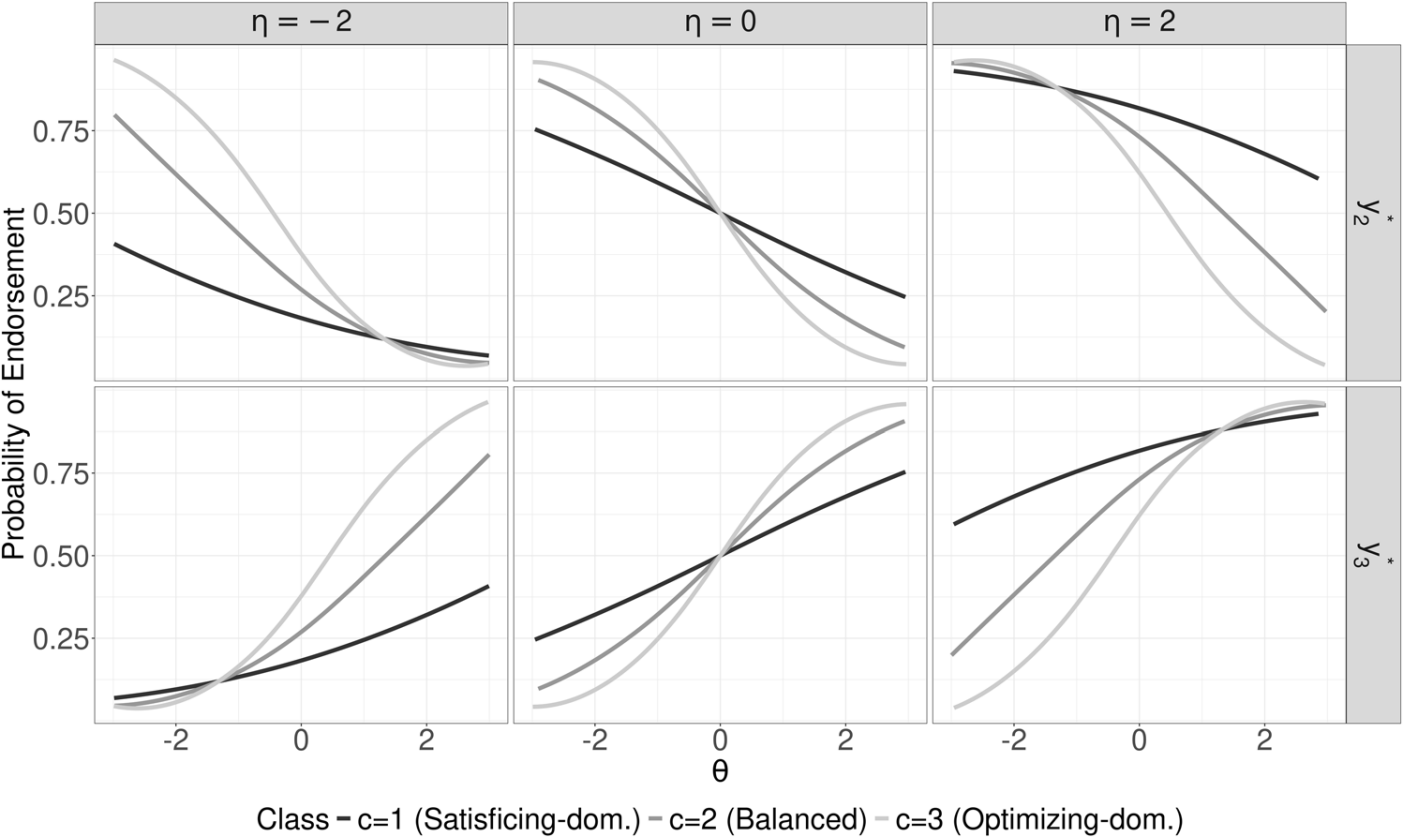
Class-specific extreme decision node probabilities as a function of trait and ERS scores for a hypothetical case, where $\beta _{1}=\beta _{2c}=\beta _{3c}=0$, $\alpha ^{(trait)}=1$, $\alpha ^{(ers)}_{c}=\{.75,.50,.25\}$, and $\omega _{c}=\{.25,.50,.75\}$

#### Hypothetical example

Let us illustrate an example scenario where there are three subpopulations, each of which is following a different response strategy. The first subpopulation follows a satisficing-dominated strategy, the second follows a balanced strategy, and the third follows an optimizing-dominated strategy. For illustrative purposes, we focus on only one hypothetical item. For brevity, assume that all node intercepts for each subpopulation are zero $(\beta _{1}=\beta _{2c}=\beta _{3c}=0)$ and the trait loading $\alpha ^{(trait)}$ of the item is one.

In the satisficing-dominated strategy, respondents mainly make use of the ERS factor, thus the proportionality constant $\omega _{1}$ is 0.25 and the ERS factor loading $\alpha ^{(ers)}_{1}$ is 0.75. In the balanced strategy, respondents make use of the trait and ERS factor to the same extent, resulting in both $\omega _{2}$ and $\alpha ^{(ers)}_{2}$ equal to 0.5. Finally, in the optimizing strategy, respondents mainly use the trait for deciding on their specific category decisions, thus $\omega _{3}$ equals 0.75 and $\alpha ^{(ers)}_{3}$ equals 0.25.^2^

Figure 2 shows probabilities of endorsing extreme decision nodes for the different classes as a function of trait scores $\theta$ and ERS scores $\eta$. In the satisficing-dominated strategy, extreme decision probabilities are strongly affected by the ERS factor as a reflection of the high $\alpha _{c}^{(ers)}$ parameter applying in this class (see between-columns trajectories). In contrast, the effect of the trait is less pronounced in line with the low $\omega _{c}$ parameter (see within-column trajectories). For the optimizing-dominated strategy, the opposite pattern is observed. For a constant trait score, extreme decision probabilities remain almost the same for different ERS scores as the ERS loading is very low, whereas a higher trait score strongly decreases the probability of the extreme disagreement choice and increases the probability of the extreme agreement choice for all levels of ERS, reflecting the high $\omega _{c}$ parameter. Lastly, in the balanced strategy, both the trait and ERS factor affect the probabilities to a similar extent in line with the specified $\alpha _{c}^{(ers)}$ and $\omega _{c}$ parameters.

Ignoring the heterogeneity for such populations would result in biased decision probabilities, whereas the proposed MixTree would successfully differentiate the class-specific probabilities by also providing correct item estimates for each class.

#### Estimation

##### Step 1

We implemented a three-step procedure to estimate the mixture model and the latent regression model capturing covariate effects on class memberships (Vermunt, 2010). In the first step, proportions of the latent classes $\pi _{c}$ and the item parameters of the MixTree are estimated by maximizing the following log-likelihood. The trait and ERS factor scores are then obtained using the expected a posteriori method. Note that in this step, the class proportions are not conditional on the covariates; thus the components of the mixture model are estimated without considering the covariate effects.

3 $$\begin{aligned} \begin{aligned} \log L_{step 1}&= \sum _{p=1}^{N} \log P(\mathbf {Y_{p}}) \\&= \sum _{p=1}^{N} \log \biggl ( \sum _{c=1}^{C} P(X=c) \times \prod _{j=1}^{J} P(y^*_{1p} | X=c) \\&\quad \times P(y^*_{2p} | X=c)^{(1 - y^*_{1pj})} \times P(y^*_{3p} | X=c)^{y^*_{1pj}} \biggr ) \end{aligned} \end{aligned}$$

##### Step 2

The second step involves using the parameter estimates from the first step to calculate posterior class membership probabilities for the respondents. Additionally, we calculate classification error probabilities as well. The latter will be needed in the third step when estimating the covariate effects on class memberships. Applying the Bayes’ rule, the posterior class membership probabilities of respondent *p* can be obtained as follows:

4 $$\begin{aligned} \begin{aligned} P(X=c|\mathbf {Y_{p}})&= \hat{\pi }_{pc} = \frac{\hat{\pi }_{c}\times P(\mathbf {Y_{p}}|{X=c})}{\sum _{c=1}^{C} \hat{\pi }_{c} \times P(\mathbf {Y_{p}}|X=c)} \end{aligned} \end{aligned}$$

Next, we use the modal assignment rule to assign respondents to latent classes. The modal assignment rule simply refers to assigning respondents to the class for which they have the largest posterior membership probability:

$$ w_{p} = argmax(\hat{\pi }_{p1},...,\hat{\pi }_{pC}) $$

For example, if we were to fit a two class MixTree model and obtain posterior class probabilities such as $\hat{\pi }_{p+}=\{0.75, 0.25\}$^3^, then $w_{p}$ would be "1", indicating the person is assigned to the first class.

Naturally, as we are operating with estimates rather than true parameter values, there is a chance of misclassification. For the hypothetical case above, we assigned a respondent to the first class, but there was still a chance that the respondent belonged to the second class with a probability of 0.25. That is, we may assign a respondent to the class *c* where the true class of the respondent is $c'$. As the true class is not known to us, we can only calculate the probability of classification errors. Since $X=c$ denotes the true class, we use $w_{p}=k$ to indicate a respondent’s assigned class membership, where $k\in \{1,...,c,...,C\}$. Then, the classification error probabilities are calculated as follows and collected in $C \times C$ matrix:

5 $$\begin{aligned} \begin{aligned} P(w_{p}=k | X=c)&= \frac{1}{N}\sum _{p=1}^{N}\frac{P(X=c|\mathbf {Y_{p}})\times P(w_{p}=k|\mathbf {Y_{p}})}{P(X=c)} \end{aligned} \end{aligned}$$

In this $C \times C$ matrix, the off-diagonal elements $c \ne k$ denote classification errors, and the diagonal elements $c=k$ denote the classification accuracy. The total proportion of classification errors can easily be obtained as follows (Vermunt, 2010):

$$ \sum _{c}^{C} P(X=c)\sum _{c \ne k} P(w_{p}=k|X=c) $$

##### Step 3

In the third step, we investigate the effects of covariates on class memberships. It can be achieved by means of a multinomial logistic regression parametrization, as denoted in Eq. 6.

6 $$\begin{aligned} \begin{aligned} {P}\left( {X=c|\mathbf {Z_p}}\right) =\frac{\exp {\left( \gamma _{0c}+\sum _{t=1}^{T}\gamma _{tc}z_{pt}\right) }}{\sum _{c=1}^{C}\exp {\left( \gamma _{0c}+\sum _{t=1}^{T}\gamma _{tc}z_{pt}\right) }} \end{aligned} \end{aligned}$$

Here $\gamma _{0c}$ is the intercept parameter for class *c*. The slope parameter $\gamma _{tc}$ is then the effect of covariate $z_{t}$ on the probability of belonging to class *c*. As true class memberships are unknown to us, a naive approach would be to replace $P(X=c)$ with the assigned class memberships $w_{p}$, and regress them on the covariate variables.

However, this approach may result in biased parameter estimates as well as inflated standard errors (Bakk et al., 2013, 2014; Bakk & Kuha, 2021; Vermunt, 2010). The reason is that we work with *estimates* from the MixTree model to calculate posterior class probabilities rather than true parameter values. Related, the class assignments contain some classification error. Therefore, replacing $P(X=c)$ with $w_{p}$ incorporates further uncertainty in the estimation.

As a remedy, Vermunt (2010) proposed using the classification errors to account for the uncertainty in class assignments as given in Eq.  7:

7 $$\begin{aligned} \begin{aligned} P(w_{p}=k | \mathbf {Z_{p}})&= P(X=c|\mathbf {Z_{p}})\times P(w_{p}=k|X=c) \end{aligned} \end{aligned}$$

The term $P(X=c|\mathbf {Z_{p}})$ already appeared in Eq. 6, and the term $P(w_{p}=k|X=c)$ is the classification (error) probabilities that appeared in Eq. 5. We can see Eq. 7 as a basic latent class model, where covariates are treated as indicators of the true class memberships, class proportions are conditioned on the covariate variables via a multinomial logistic regression model, and classification error probabilities are treated as fixed conditional response probabilities that weigh and correct for the association between $P(X=c)$ and $\mathbf {Z_{p}}$. Readers interested in further details of the procedure are referred to Vermunt (2010), Bakk et al. (2013, 2014), and Alagöz and Vermunt (2022).

All of the above steps can easily be conducted in the R software. In this study, the estimation of the MixTree model with no covariates, which is the first step, is conducted with the ’mirt’ package.

#### Model selection

The model selection procedure for the mixture models plays an important role for two reasons. First, we want to detect whether heterogeneity exists or not. Second, if we find evidence that heterogeneity may exist (i.e., in the case when the model with one-class is not favored) we want to find the correct class enumeration (i.e., number of latent classes). For this reason, we computed several model selection criteria as follows:

$$ AIC = -2\log L_{step1} + 2\times n_{k}\, (\text {Akaike}, 1974)$$

$$ BIC = -2\log L_{step1} + \log N \times n_{k}\, (\text {Schwarz}, 1978)$$

$$ HBIC = -2\log L_{step1} + n_{k_{o}}\log (N) + \sum _{c=1}^{C} n_{k_{c}}\log (\pi _{c}N)\, (\text {Zhao et al.}, 2015)$$

Above $n_{k}$ is the total number of freely estimated parameters, which is the sum of freely estimated class-invariant parameters $n_{k_{o}}$ and freely estimated class-specific parameters $n_{k_{c}}$:

$$ n_{k} = n_{k_{o}} + n_{k_{c}}$$

$$ n_{k_{o}} = 2 \times J + 1 + (C-1)$$

$$ n_{k_{c}} = C \times (3 \times J + 1)$$

## Simulation study

We conducted an extensive simulation study to investigate the performance of the MixTree model for the recovery of parameters under different numbers of classes, different class sizes, different numbers of items and sample sizes, and varying covariate effects. We also investigated how the separation between classes or the correlation between the trait and ERS affects the performance. In addition to investigating bias in parameter estimates, we explored how different information criteria perform in model selection and class enumeration.

### Design

We use a hypothetical population where respondents can follow one of three types of response strategies. Each of these strategies refers to nodes two and three thus to gradual decision within (dis)agreement categories similar to those implemented in Kim and Bolt (2021). The first strategy is a satisficing-dominated process where respondents mainly use ERS while deciding on their category choices. The second strategy involves similarly weighted use of the trait and ERS, and the last one is an optimizing-dominated strategy where the trait is weighted dominantly in the response strategy.

**Table 2:** Number of classes (*C*) and respective class size conditions

C	$\bar{\pi }_{+1}$	$\bar{\pi }_{+2}$	$\bar{\pi }_{+3}$
1	1	0	0
	0	1	0
	0	0	1
2	0.5	0.5	0
	0.5	0	0.5
	0	0.5	0.5
3	0.33	0.33	0.33
	0.6	0.2	0.2
	0.2	0.6	0.2
	0.2	0.2	0.6

*Note.* Class probabilities were obtained by plugging in covariate data and regression parameters in Eq. 6. The covariate data is randomly sampled in each simulation replication, causing small deviations (± 0.05 to ± 0.10) from the presented values

First, we manipulated the number of classes at three levels, ${C}=\{1, 2, 3\}$. Second, we manipulated the size of classes for each level of *C* (see Table 2). Third, we manipulated the number of items and respondents at two levels, ${J=\{10, 20, 30\}}$ and ${N=\{1000, 2000, 3000\}}$. We fixed the number of covariates at $T=3$. We were also interested in the recovery of covariate effects for different effect sizes. We investigated the recovery by setting different effect sizes for each covariate rather than manipulating them at the between-conditions levels. Specifically, the first covariate has a strong effect on class membership, the second one has a weak effect, and the third one has no effect on class memberships (see Table 3). The trait and the ERS factor were sampled from a multivariate normal distribution, where their means were fixed at zero, variances were fixed at 1, but their correlations were either zero or 0.30. Finally, for $C>1$, we manipulated the separation between classes to be low $(R_{entropy}^{2}\approx 0.20)$, medium $(R_{entropy}^{2}\approx 0.40)$ or high $(R_{entropy}^{2}\approx 0.70)$. For each cell of the simulation design, we generated 200 data sets, resulting in $7 \times 3 \times 3 \times 2 \times 3 \times 200 = 75600$ data sets from a mixture population and $3 \times 3 \times 3 \times 2 \times 1 \times 200 = 10,800$ data sets from a non-mixture population, together, 86, 400 data sets to be analyzed.

**Table 3:** Covariate effects for three equal classes condition

Parameters	Classes		
	1 (ref)	2	3
$\gamma _{0}$	0	-0.37$^{\text {a}}$	-0.37$^{\text {a}}$
$\gamma _{1}$	0	-1.00	1.00
$\gamma _{2}$	0	0.50	-0.50
$\gamma _{3}$	0	0	0

Note. For conditions with two classes, the last column was removed and the remaining parameters were used

$^{\text {a}}$. The slope parameters were kept constant and the intercept parameters were manipulated to obtain different class sizes in Table  2

**Table 4:** The distributions that were used in sampling item parameters for the class-specific parameters at nodes two and three based on the class separation levels

Parameter	Separation	Classes		
		1 (Satisficing-dominated)	2 (Balanced)	3 (Optimizing-dominated)
$\omega _{jc}$	Low	$\omega _{j2}$ – U(0, 0.1)	U(0.5, 0.7)	$\omega _{j2}$ + U(0, 0.1)
	Medium	$\omega _{j2}$ – U(0.2, 0.3)	U(0.5, 0.7)	$\omega _{j2}$ + U(0.2, 0.3)
	High	$\omega _{j2}$ – U(0.4, 0.5)	U(0.5, 0.7)	$\omega _{j2}$ + U(0.4, 0.5)
$\alpha ^{(ers)}_{c}$	Low	$\alpha ^{(ers)}_{2}$ + U(0, 0.1)	U(0.5, 0.7)	$\alpha ^{(ers)}_{2}$ – U(0, 0.1)
	Medium	$\alpha ^{(ers)}_{2}$ + U(0.2, 0.3)	U(0.5, 0.7)	$\alpha ^{(ers)}_{2}$ – U(0.2, 0.3)
	High	$\alpha ^{(ers)}_{2}$ + U(0.4, 0.5)	U(0.5, 0.7)	$\alpha ^{(ers)}_{2}$ – U(0.4, 0.5)
$\beta _{2jc}$	Low	$\beta _{2j2}$ + N(0, 0.25)	N(0, 1)	$\beta _{2j2}$+ N(0, 0.25)
	Medium	$\beta _{2j2}$ + N(0, 0.75)	N(0, 1)	$\beta _{2j2}$+ N(0, 0.75)
	High	$\beta _{2j2}$ + N(0, 1.5)	N(0, 1)	$\beta _{2j2}$+ N(0, 1.5)
$\beta _{3jc}$	Low	$\beta _{3j2}$ + N(0, 0.25)	N(0, 1)	$\beta _{3j2}$+ N(0, 0.25)
	Medium	$\beta _{3j2}$ + N(0, 0.75)	N(0, 1)	$\beta _{3j2}$+ N(0, 0.75)
	High	$\beta _{3j2}$ + N(0, 1.5)	N(0, 1)	$\beta _{3j2}$+ N(0, 1.5)

*Notes:*

*1.* The terms added to the intercept parameters for the first and third classes are used to control class separation, i.e., the degree of distinction between the response models of each class. The specific standard deviation parameters for the normal distributions were determined through a pilot study, in which we explored which values yielded the desired $R^2$ entropy levels

*2.* Since the added terms for the intercepts in the first and third class may cause extreme values, we further truncated the intercept terms so that they stay between the range of -3 and 3

*3.* We draw different item parameters at each simulation replication from the distributions given in the table so that the simulation study is highly generalizable as it captures a broad range of potential parameter values and their combinations

*4.* The choice for distribution of the $\omega _c$ parameter was informed by empirical findings from previous studies (Alagöz & Meiser, 2024; Meiser et al., 2019). Then, $\alpha _{c}^{(ers)}$ is sampled to reflect the chosen response strategies. The empirical findings later showed that our choices were in line with the empirical data set.

Below, we describe the data generation process along with the choice of parameter values.

1. T=3 number of covariates, $Z_{+p}$, were sampled for N number of respondents from $$\sim MVN\left( \textbf{0}, \begin{bmatrix} 1 & 0 & 0\\ 0 & 1 & 0\\ 0 & 0 & 1 \end{bmatrix}\right) $$
2. For $C>1$ conditions, the fixed $\gamma$ parameters and the covariates $Z_{+p}$ were plugged in Eq. 6 to calculate class membership probabilities $\pi _{p+}$ for each respondent *p*.
3. The true class membership for each respondent was drawn from a multinomial distribution using the class membership probabilities $\pi _{p+}$.
4. The trait and ERS scores were sampled at each replication from $$MVN\left( \textbf{0}, \begin{bmatrix} 1 & \sigma \\ \sigma & 1 \end{bmatrix}\right) $$, where $\sigma$ is "0" or "0.30" depending on the correlation level of the simulation design.
5. The class-invariant item parameters at the first node, $\alpha ^{(trait)}_{j}$ and $\beta _{1j}$, were sampled at each replication from *U*(0.5, 1.25) and *N*(0, 1).
6. The class-specific item parameters at the second and third nodes are sampled from the distributions presented in Table 4, depending on the separation level of the simulation design.
7. By plugging the sampled person and item parameters into the MixTree model equation, we calculated the category probabilities per person and item. Then the actual responses were sampled from a multinomial distribution, resulting in $N \times J$ data matrix.
8. The $N \times J$ data matrix was decomposed into pseudo-items by following the rules in Table 1, resulting in $N \times 3J$ data matrix to be fit with the MixTree model.
9. For $C=1$ conditions, the first step was also applied, meaning that three covariates were sampled from the same multivariate normal distribution. However, they were not associated with class memberships as in the second and third steps. Therefore, the class memberships were not sampled by calculating probabilities but rather fixed to "1", "2", or "3" depending on the class proportion condition presented in Table  2. Then, steps 4 to 8 were followed, with one difference. That is, only the middle rows (i.e., *medium separation*) were used since class separation does not apply in single-class populations.

The steps above were repeated to generate 86,400 data sets in total. We fitted three models to each generated data set, by increasing the estimated latent classes from one to three, the MixTree-1 (i.e., the traditional single-class IRTree), MixTree-2, MixTree-3, respectively. For the estimation in the simulation study, we used four random starts to fit the MixTree-1 and eight random starts to fit the MixTree-2 and MixTree-3 models. We then proceeded with the solution that resulted in the largest $\log L_{step1}$. We then examined whether the label-switching phenomenon occurred. Since the likelihood is invariant to class labels, label assignments can be switched arbitrarily across estimation runs with different starting values. When label switching was detected, we realigned the estimated class labels to match the true label ordering used in the data-generating process, enabling meaningful comparisons in subsequent analyses. Such realignment is unnecessary in empirical applications, where the true class ordering is unknown and thus no direct comparison is required. Finally, note that the MixTree model does not restrict the total number of latent classes or does not predefine any latent class as optimizers or satisficers. Unlike in Alagöz and Meiser (2024); Kim and Bolt (2021); Tijmstra et al. (2018), all item parameters are freely estimated and the number of classes can be determined via model selection procedure. Therefore, for the empirical data analysis, one can fit even larger number of classes and should interpret the class definitions post hoc.

**Fig. 3:**
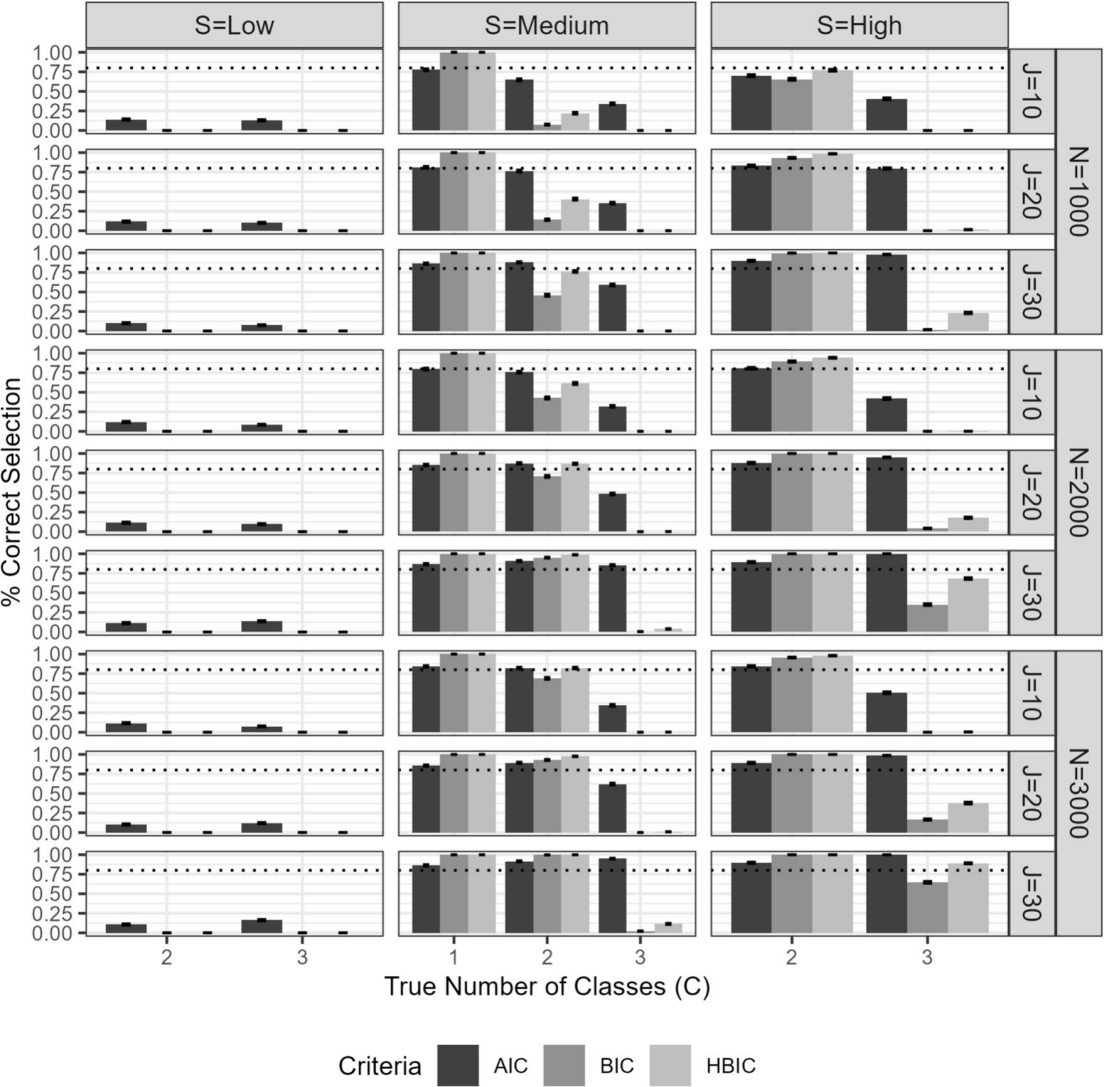
The proportion of correctly selected model among MixTree-1, MixTree-2, and MixTree-3 across different sample sizes (N), test lengths (J), class separations (S), and class proportions. The *dashed line* is the reference point for 0.80

### Results

In this section, we present the results of the simulation study. We merged the levels of the correlation condition as the results were indifferent for when the correlation between the trait and ERS was zero and when it was 0.30^4^. Furthermore, we also merged class proportion levels given a number of class for brevity and to facilitate the interpretation. We used mean bias and root mean squared error (RMSE) to assess parameter recovery. Specifically, mean bias is investigated to see if there is a systematic under- or over-estimation of a parameter, whereas RMSE is used to assess the accuracy of estimates while taking the uncertainty into account.

$$\overline{Bias}= \frac{1}{N_{rep}}\sum _{rep=1}^{N_{rep}} (\hat{\lambda }_{rep}-\lambda _{rep})$$

$$RMSE= \sqrt{\frac{1}{N_{rep}}\sum _{rep=1}^{N_{rep}} (\hat{\lambda }_{rep}-\lambda _{rep})^{2}}$$

where, $\lambda _{rep}$ is the parameter of interest at a replication, $\hat{\lambda }_{rep}$ is the point estimate of parameter $\lambda$ at the same replication.

#### Model selection

Figure 3 presents the model selection performance of the AIC, BIC, and HBIC criteria across varying sample sizes (N), test lengths (J), and class separations (S). Overall, the results demonstrate that the AIC outperformed BIC and HBIC in correctly identifying the true number of latent classes, particularly when class separation and test length were adequate.

The results reveal that class separation was a key factor influencing model selection accuracy. In the low separation condition, the latent classes overlapped substantially, resulting in similar measurement models across classes and reducing the ability of the MixTree model to distinguish between classes. Under these conditions, all criteria failed to correctly identify the number of classes regardless of the test length.

In the medium separation condition, a clearer pattern emerged. When C=1, indicating homogeneity in the population, all criteria successfully rejected the need for additional classes across test lengths and sample sizes (note that C=1 condition is always presented under medium separation, as there cannot be class separation for a single-class population). For conditions with C=2, the AIC showed consistently high accuracy, with correct model selection rates exceeding 0.80 in most cases, except under the shortest test (i.e., J=10) and smallest sample size (N=1000) conditions. In contrast, the BIC and HBIC required larger sample sizes and longer tests to achieve comparable accuracy, with performance improving only under conditions of N=2000 or larger and J=20 or longer. When C=3, the AIC maintained reasonable accuracy given a large sample size and long test, but BIC and HBIC continued to perform poorly regardless of sample size or test length.

In the high separation condition, all criteria performed well in identifying the correct model when C=2 across most test lengths and sample sizes, highlighting the robustness of the criteria when classes are well separated. For C=3, the AIC continued to demonstrate high accuracy across most conditions except under the shortest test length (J=10) condition. In comparison, BIC and HBIC required the longest test length (J=30) and the largest sample size (N=3000) to successfully detect the three-class structure, indicating that these criteria are more sensitive to increased sample size and test length under conditions of high separation.

In summary, the AIC outperformed both BIC and HBIC in accurately detecting heterogeneity and identifying the correct number of latent classes, particularly under realistic conditions of moderate class separation and sufficient test length. Given the limitations observed for BIC and HBIC in conditions of low separation or short tests, we recommend that researchers use AIC as the primary criterion for model selection when conducting analyses with MixTree. However, especially when class separation is low or test length is limited, researchers should consider supplementing AIC-based selection with the $R^{2}_{entropy}$ statistic to assess model selection.

#### Classification

We wanted to ensure that the MixTree performs well for classifying respondents in their true latent class. To assess the recovery of class assignments, we calculated the hit rate (HR) as the proportion of respondents who were assigned to their true classes.

**Fig. 4:**
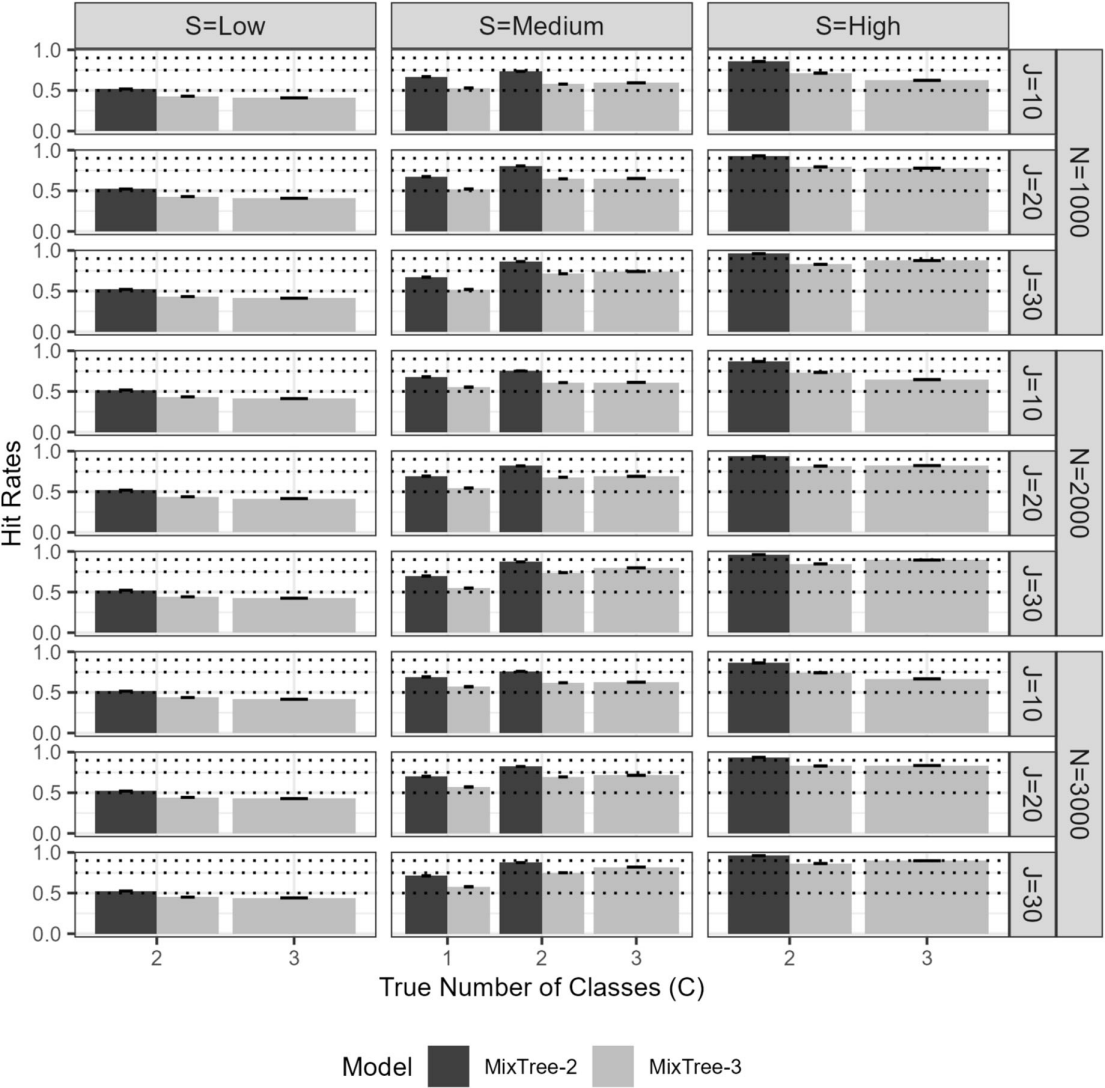
The proportion of respondents assigned to correct latent class by MixTree-2 and MixTree-3 across different sample sizes (N), test lengths (J), class separations (S), and class proportions. The *dashed lines* are reference points for 0.50, 0.75, 0.90

**Fig. 5:**
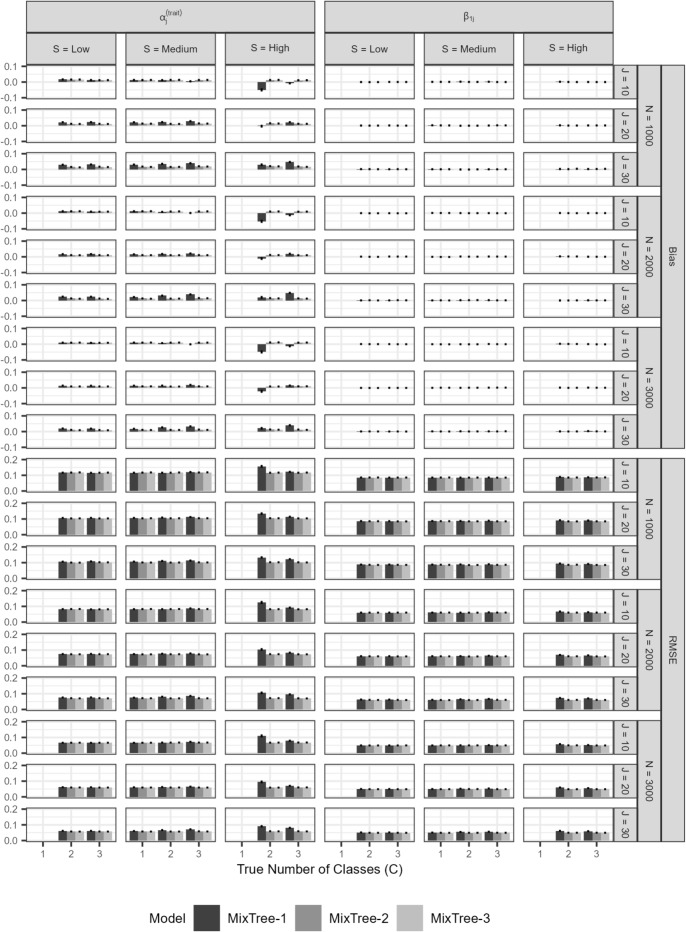
Bias and RMSE of class-invariant parameter estimates of $\alpha _{j}^{(trait)}$ and $\beta _{1j}$ at the first node obtained with MixTree-1 to -3, across different sample sizes (N), test lengths (J), class separations (S), and true number of latent classes. Note that the *Y*-axis scales are different on each row

As is seen in Fig. 4, the MixTree performed well in class assignments when the class separation was not low or test length was not short. For the effect of class separation, we found that the classification accuracy was highest when class separation was medium or high, with hit rates spanning from 0.75 to 0.95 for $C>1$ and approaching 0.75 for C=1 as a function of test length. Under low separation, the classification accuracy was substantially reduced as shown by lower hit rates around 0.50 across all test length, sample size conditions for both MixTree-2 and MixTree-3 models. This finding highlights that increased separation between latent classes is critical for achieving high classification accuracy, especially when the test length is limited.

We also found that the discrepancy between the population and model complexity affected the classification performance. Specifically, when the true population structure is relatively homogeneous than the fitted MixTree model (e.g., fitting MixTree-3 in a population with one or two classes), the simpler MixTree-2 model outperformed the MixTree-3 model in classification accuracy. The added complexity of MixTree-3, with an extra latent class, led to increased classification uncertainty due to the capture of noise in the additional class. This effect was particularly pronounced in shorter tests or with low separation but was mitigated as test length increased, suggesting that longer tests may reduce the noise and provide additional information that helps more complex models in classification. As we also implemented in the *Empirical Illustration*, one can conduct further techniques such as cross-validation to ensure that the emerged classes carry substantive information rather than noise.

The sample size condition had only a negligible positive effect on classification. The correlation between factors did not make a difference in the performance, so they are merged in Fig. 4.

In summary, the MixTree models demonstrate strong classification performance under realistic conditions, provided that the class separation is not low and the test length is sufficient. Researchers are recommended to pay attention to separation between classes and their test’s length when interpreting class assignments. To prevent the risk of overfitting, which may cause discrepancy between true and fitted complexity, particularly when aiming to use class assignments in further analyses, it is recommended to apply multiple random starts and stricter convergence criteria in the expectation-maximization (EM) algorithm. Additionally, model selection results indicate that the AIC generally safeguards against under- or overfitting, except in extreme cases with very short tests and low separation.

#### Recovery of item parameters

##### Class invariant item parameters

In Fig. 5, we present the mean bias and RMSE of the class-invariant parameters at the first node, namely, $\alpha _{j}^{(trait)}$ and $\beta _{1j}$ averaged across J. The scales on the *y*-axis for bias range from -0.05 to 0.05, implying that there is only a very small amount of bias in the parameter estimates across all conditions.

Specifically, when we investigate the left panel of the top half, we see that a MixTree model with more than one class, usually recovers the trait loadings at the first node without being affected by the class separation, test length, and sample size conditions. When falsely fitted to a mixture population, the MixTree-1 resulted in slightly increased bias in the $\alpha _{j}^{(trait)}$ parameter.

In the bottom half, the RMSE values are generally small and very similar across conditions. The only exception is when the class separation is high, where fitting a MixTree-1 model to a more complex population leads to increased RMSE values, implying lower accuracy of the $\alpha ^{(trait)}_{j}$ estimates. Otherwise, the RMSE were found to decrease as the test length, sample size, and class separation increase.

On the right panel of the top half of the figure, we see that the bias in the intercepts were consistently almost zero across all conditions and different MixTree models. It is an expected result as it is generally easier to recover intercept parameters with even very small sample sizes.

In the bottom half, we see a similar pattern as in the trait factor loadings. That is, fitting MixTree-1 to a more complex population led to slightly increased RMSE values. Furthermore, with an increasing test length, sample size, and class separation, the RMSE values consistently decreased.

##### Class-specific item parameters

**Fig. 6:**
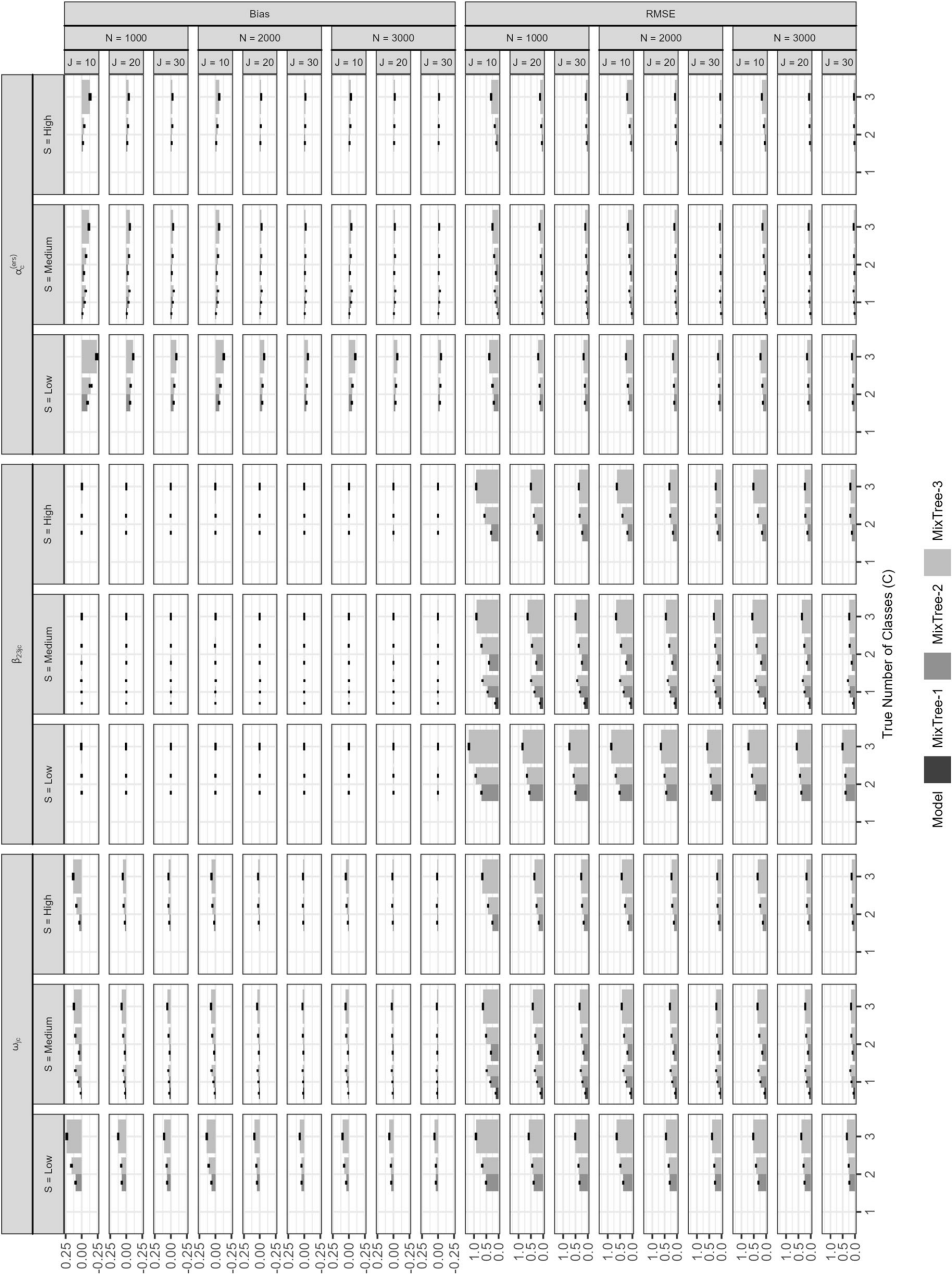
Bias and RMSE of class-specific parameter estimates of $\omega _{jc}$, $\alpha _{c}^{(ers)}$ and $\beta _{23c}$ at the second and third node obtained with MixTree-1 to -3 across the sample size (N), test length (J), class separation (S), and true number of classes. Note that *Y*-axis scales are different on each row

In Fig. 6, we present the results for the class-specific item parameters at nodes two and three. Specifically, we present the average bias in estimates obtained with MixTree-1 to -3 across different sample sizes (N), test lengths (J), and true number of classes (C).

$\omega _{jc}$: On the left-most panel of the top half, we present the bias in proportionality constants averaged across items. As in other cases, class separation was the most prominent factor affecting the recovery, which was followed by test length. More specifically, under low class separation, proportionality constants were over-estimated, but the extent of bias seemed to diminish as mainly with increasing test length but also slightly with increasing sample size. The same pattern was also observed for the medium and high separation conditions, but the overall bias was much lower than in the low class separation condition.

On the left-most panel of the bottom half, we present the RMSE values for $\omega _{jc}$. The RMSE values were the largest for the smallest test length and lowest separation condition. Furthermore, they were overall very small, also when compared to the other class-specific parameters, likely because the class-invariant involvement of the trait across classes, which improves the information regarding all trait-relevant parameters.

$\beta _{2jc}$ and $\beta _{3jc}$: On the middle panel of the top half, we present the bias in the class-specific intercept terms averaged across items and nodes (i.e., $\beta _{23jc}$ in Fig. 6), for which we did not observe any salient over- or under-estimation. That is, as in the class-invariant intercept parameter of node one, all class-specific intercept parameters were recovered without any bias in all conditions that we considered.

When the RMSE values on the bottom-half are investigated, we see rather high values for the lowest test length, sample size, and separation conditions. Yet, the accuracy of estimates improves drastically as the test length, sample size, and class separation increases.

$\alpha ^{(ers)}_{c}$: On the right-most panel, we provide the bias of the class-specific ERS factor loading estimates. We found almost the same pattern as the results for the proportionality constants with one difference, that is, the ERS factor loading was associated with negative bias, meaning under-estimation. This under-estimation was most visible in the low class separation. However, under all separation conditions, the negative bias greatly reduced as the test length and sample size increased.

On the bottom half, we present the RMSE values. We found a similar pattern as for other parameters. That is, the lowest test length, sample size, and class separation conditions yield the largest RMSE values, but it decreases significantly with a longer test, larger sample size, and higher class separation.

#### Person parameters

**Fig. 7:**
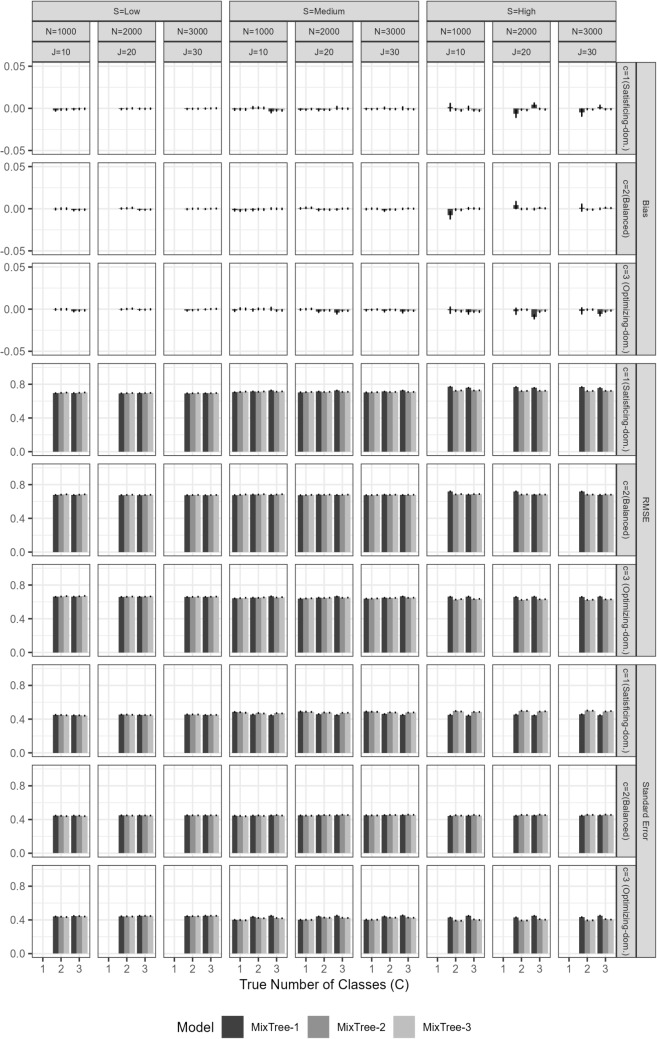
Bias, mean squared error, and mean standard errors of trait ($\theta$) estimates obtained with MixTree-1 to -3 separately for each existing class across different sample sizes (N), test lengths (J), class separations (S), and true number of classes

**Fig. 8:**
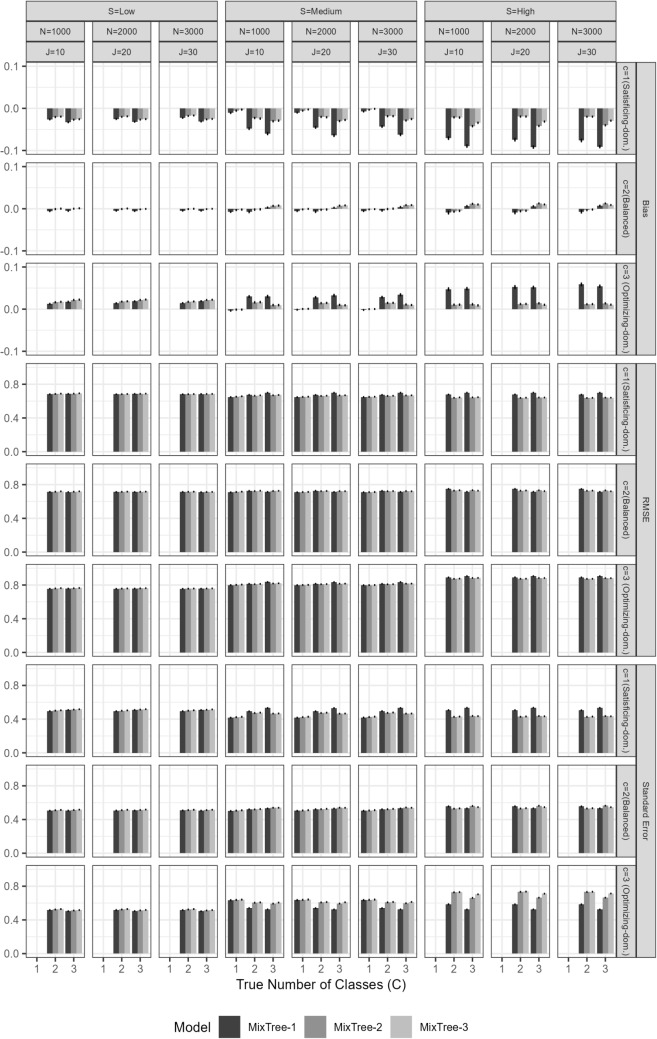
Bias, mean squared error, and mean standard errors of ERS ($\eta$) estimates obtained with MixTree-1 to -3 separately for each existing class across different sample sizes (N), test lengths (J), class separations (S), and true number of classes

Figures 7 and 8 show the recovery of trait ($\theta$) and ERS $\eta$ scores using MixTree models with varying numbers of classes (1, 2, or 3) under different simulation conditions: true number of classes, class separation (S), sample size (N), and test length (J). The three columns of panels depict mean bias, root mean squared error (RMSE), and mean estimated standard error (SE) across simulation conditions. The latter, mean estimated standard error, is calculated in order to evaluate the precision of estimated factor scores.

##### Recovery of the trait scores $\theta$

The first three row of the top panel present the mean bias in trait score estimates separately for each class, where the bias was averaged across the members of each class. The first row demonstrates the bias for respondents in the satisficing-dominated class, where respondents put less weight on the trait than the ERS factor in their response strategies, the second row for those who used both the trait and ERS to a similar extend, and the third row for the optimizing-dominated strategy, where the trait played a stronger role than the ERS in the response strategies. In almost all conditions, we found near-zero bias in the trait scores with an exception under the high separation condition. Under high separation, some conditions showed a slight under- or over-estimation of trait scores by the MixTree-1 model. However, given that the mean bias for these conditions were ranging between -0.01 and 0.01 (note the scale of the *y*-axis), we consider them negligible and do not interpret any further.

In the middle panel, we present the RMSE values, again, for each class separately. Similar to the bias results, we found that all models yielded similar RMSE values regardless of the condition and the true number of classes in the population, except the high separation condition. Under high class separation, we found that MixTree-1 yielded slightly higher RMSE for each class than MixTree-2 and MixTree-3 when the population consisted of more than one class.

In the bottom panel, we present the results for the mean estimated standard errors separately for each class. Again, the mean SE estimates were comparable between all MixTree models under all conditions but the high separation. When class separation was high, the MixTree-1 model, in comparison with MixTree-2 and -3 models, reported the SE of trait scores in optimizing-dominated class slightly larger and in satisficing-dominated class slightly smaller.

##### Recovery of the ERS scores $\eta$:

At the top panel, we present the results for the mean bias of ERS score estimates separately for each class and averaged across respondents within each class. The first row is the subset of respondents who belong to the satisficing-dominated strategy class, where the relative weight of the ERS was greater than the trait. The results show consistent underestimation of ERS scores by all MixTree models. However, the bias was even larger for the MixTree-1 model under $C>1$ conditions, and it got even larger with the increasing class separation. Under $C=1$ condition, however, all three models yielded ignorable bias. Furthermore, the results suggest neither the test lengths nor the sample size had a substantial effect on the bias in ERS score estimates.

The second row is the subset of respondents who belong to the balanced strategy class, where the trait and ERS were roughly equally weighted. For respondents belonging to the balanced class, we found only ignorable bias (i.e., near-zero) in the ERS scores across all test length, separation, and sample size conditions.

The third row is the subset of respondents who belong to optimizing-dominated strategy class, where the ERS had less weight than the trait. For those respondents, we found the opposite pattern of the first class. That is, the ERS scores were systematically over-estimated across all conditions, but the extent of over-estimation increased further with a higher class separation.

In the middle panel, we present the RMSE values of the ERS scores, again, for each class separately. As the figure suggests, the RMSE values were consistent across different classes, but slightly increased for the MixTree-1 model under $C>1$ and high class separation conditions, implying the poor performance due to unaccounted heterogeneity, whereas MixTree-2 and MixTree3 yielded smaller RMSE values under these conditions.

Finally, the bottom panel presents the results regarding the estimated standard errors for the ERS score estimates averaged across *N* respondents. When there was no heterogeneity ($C=1$) or the class separation was low, all MixTree models yielded similar mean SE of trait scores. However, under a heterogeneous population ($C>1$) with medium to high separation, MixTree-1 yielded the mean SE higher for satisficing-dominated class and lower for optimizing-dominated class than MixTree-2 and -3. For the balanced strategy class, the mean estimated SE were comparable.

In conclusion, when a traditional IRTree model was fit in a population where sub-populations use qualitatively different response strategies, it did not result in a systematic under- or over-estimation of trait scores, but the RMSE values and mean estimated SEs implied worse accuracy. The reason for the good recovery of the trait scores with all MixTree models, regardless of the population heterogeneity is the class-invariant first node, making the model gather adequate information about the trait regardless of a potential model misspecification at the later class-specific nodes.

We found, however, that the ERS scores recovered poorly by the MixTree-1 model if the population consisted of multiple classes. Specifically, respondents who placed less weight on the ERS factor in their response strategy (i.e., optimizing-dominated class) had their ERS scores overestimated, while those who placed greater weight on the ERS(i.e., satisficing-dominated class) had their trait scores underestimated. Notably, no systematic bias was observed for the balanced class. These findings suggest that MixTree-1 produced parameter estimates that were averaged across all sub-populations, causing the ERS estimates to shrink towards mean of all classes. Furthermore, although this unaccounted heterogeneity was reflected as increased precision of ERS score estimates (i.e., smaller SE) for respondents in the optimizing-dominated class, it was reflected as reduced precision (i.e., larger SE) for those in the satisficing-dominated class. That is, the MixTree-1 model reported a higher precision for substantially under-estimated ERS scores.

**Fig. 9:**
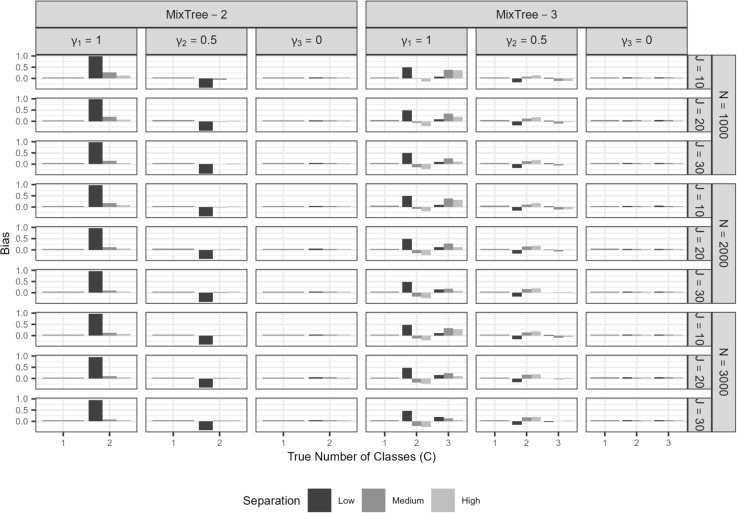
Bias in estimates of covariate effects by the MixTree-2 and MixTree-3 models across different sample sizes (N), test lengths (J), separation levels (S), and number of classes (C)

#### Recovery of covariate effects

**Fig. 10:**
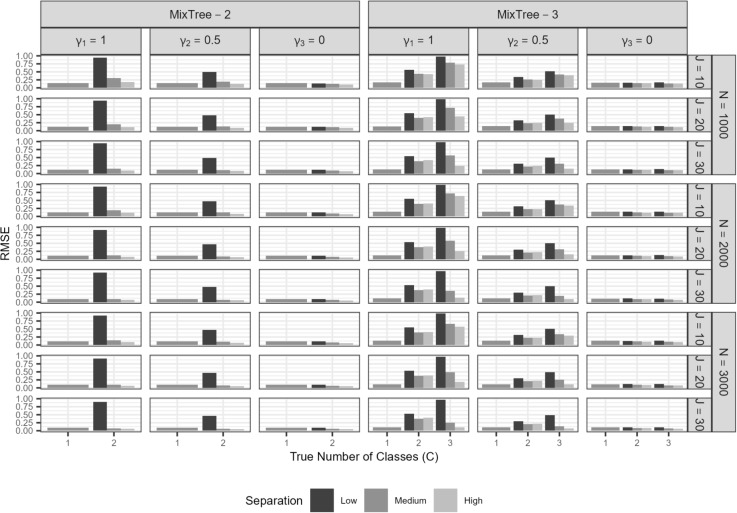
RMSE of estimates of covariate effects by the MixTree-2 and MixTree-3 models across different sample sizes (N), test lengths (J), separation levels (S), and number of classes (C)

The overall performance of the MixTree regarding the recovery of covariate effects was very good except for the extremely difficult conditions (see Figs. 9 and 10). More specifically, there were two general patterns for the recovery performance. First, the bias and RMSE of covariate effect estimates tended to increase with stronger covariate effects. This trend aligns with the well-known phenomenon of separation bias in multinomial logistic regression, where a large covariate effect leads to high separation between outcome categories. This high separation means that certain predictor values can almost perfectly predict outcome categories, akin to a steep sigmoid curve where a cutoff yields near-perfect category separation (see Albert & Anderson, 1984; Lesaffre & Albert, 1989; Zorn, 2005, for further details). This phenomenon often inflates standard errors and introduces bias into the estimates.

Second, a lower class separation also led to increased bias and RMSE of covariate effect estimates. Under low separation conditions, classes became more indistinct, leading to reduced accuracy in the MixTree’s posterior class membership probabilities. This, in turn, affected the performance of covariate effect estimation, as the model struggled to distinguish between latent classes and make accurate classifications.

When we investigate the results regarding the separation in more details, we see that the mean bias was almost equal to the true parameter value in the low class separation condition. This finding implies that despite the non-zero true values used in data generation, classes were extremely similar to each other that the impact of covariates in generating class memberships has disappeared. In other words, despite using non-zero effect sizes, the similarity between classes caused them to be practically zero.

Third, we found that, except under low class separation, increasing the test length reduced the bias remarkably. In the cases of medium separation, there was still a slight over- or under-estimation, but it can be considered negligible with a large enough sample size ($N>1000$) and long enough test length ($J>10$).

Fourth, the RMSE results show another important factor determining the recovery performance, the model complexity. As the number of fitted class increases, the RMSE values increase as well. This is rather an expected result, as the increased model complexity increases the ambiguity with the additional classes. This, in return, reduces the accuracy in the class probabilities and class assignments as shown in the results for classifications. However, such adverse effect of model complexity diminishes with the increasing sample size and test length, showing the importance of having an adequate number of items and persons in the data set for fitting the MixTree model.

## Empirical illustration: Baron-Cohen’s systemizing quotient test

The Empathizing and Systemizing Quotients Test (E-S Test) measures individuals’ empathic thinking and systemic thinking abilities (Baron-Cohen et al., 2003; Baron-Cohen & Wheelwright, 2004). Empathic thinking refers to communication skills and social interaction types such as understanding other’s perspectives, constructive conflict resolutions, and compassionate decision-making strategies, whereas systemic thinking skills are more relevant for understanding of systems, recognition of patterns, and analyzing rules and guides. The empathizing and systemizing subscores obtained from the E-S Test are then compared to investigate individuals’ differences in thinking styles, and even sometimes to examine Autism Spectrum Disorders (Baron-Cohen et al., 2003). As our aim is only to illustrate the MixTree model, we focus on the systemizing part of the test.

For our analysis, we use the open-access data set obtained from Open Psychometrics^5^. The data set contains responses from a total of 13.256 individuals to 60 items measuring systemizing abilities (Cronbach’s $\alpha =0.90$). We randomly chose a subset of 20 items with varying item-total correlations between 0.35 and 0.71 $({M}=0.52, {SD}=0.11)$, resulting in Cronbach’s $\alpha$ of 0.89.

### Model selection

We fit the MixTree model with one to four classes. According to the AIC and BIC, the best balance between model fit and complexity was achieved by the MixTree-4 and MixTree-3, respectively (Table 5). However, first, the difference between information criteria values is very small given the number of freely estimated parameters and the sample size. Second, $R^{2}_{entropy}$ values show only negligible improvement with the increasing number of fitted classes, which may be hinting at over-fitting for MixTree-3 and MixTree-4 models. Third, we investigated the probability of classification accuracy across classes (i.e., mean of the diagonal elements of Eq. 5) and found it 0.81 for MixTree-2, 0.72 for MixTree-3, and 0.66 for MixTree-4. This finding implies that the added classes incorporate large ambiguity and were likely capturing some noise in the data. Given the suspicion of overfitting, we investigated the absolute fit by means of K-folds cross-validation, which is sensitive to overfitting and can help us understand if the additional classes do not contain substantive information but rather capture noise.

**Table 5:** The AIC, BIC, number of estimated parameters, reduction in entropy, and estimated class sizes for the fitted MixTree model with number of classes from one (C=1) to four (C=4)

C	AIC	BIC	($n_{k}$)	$R^{2}_{entropy}$	$\hat{\bar{\pi }}_{+1}$	$\hat{\bar{\pi }}_{+2}$	$\hat{\bar{\pi }}_{+3}$	$\hat{\bar{\pi }}_{+4}$
1	597551	598315	102	–	1	–	–	–
2	594719	595947	164	0.41	0.53	0.47	–	–
3	593963	595656	226	0.42	0.35	0.29	0.36	–
4	593854	596001	288	0.42	0.23	0.24	0.31	0.22

**Table 6:** Comparison of item, person, and classification similarities across 5 K-folds

K Fold	Items		Factors				Classification.		
			Trait		ERS				
	Cor.	Dist.	Cor.	$|\text {Dist.}|$	Cor.	$|\text {Dist.}|$	Cor	Hit	Cohen’s $\kappa$
MixTree-2									
1	0.985	0.035	0.999	0.048	0.997	0.052	0.94	0.911	0.815
2	0.81	0.026	0.999	0.056	0.996	0.067	0.937	0.871	0.753
3	0.988	0.002	0.999	0.03	0.997	0.058	0.957	0.929	0.857
4	0.816	–0.018	0.999	0.043	0.996	0.06	0.916	0.89	0.782
5	0.801	–0.03	0.999	0.038	0.996	0.054	0.945	0.908	0.772
Mean	0.880	0.003	0.999	0.04	0.996	0.05	0.939	0.902	0.800
SD	0.097	0.028	0.001	0.01	0.001	0.006	0.015	0.022	0.041
MixTree-3									
1	0.691	0.035	0.997	0.06	0.962	0.185	0.23	0.343	0.386
2	0.824	–0.083	0.995	0.075	0.96	0.18	0.39	0.356	0.208
3	0.892	–0.027	0.995	0.068	0.976	0.143	0.466	0.259	0.182
4	0.797	–0.039	0.997	0.062	0.965	0.176	0.41	0.253	0.253
5	0.869	–0.066	0.996	0.067	0.962	0.168	0.439	0.276	0.311
Mean	0.815	–0.036	0.996	0.066	0.965	0.17	0.387	0.297	0.268
SD	0.078	0.045	0.001	0.006	0.006	0.016	0.09	0.048	0.082
MixTree-4									
1	0.734	0.019	0.997	0.059	0.972	0.156	–0.099	0.768	0.112
2	0.823	–0.063	0.996	0.061	0.963	0.19	–0.114	0.764	0.070
3	0.755	–0.049	0.997	0.059	0.97	0.154	0.422	0.211	0.001
4	0.798	–0.029	0.996	0.064	0.968	0.145	0.478	0.291	0.117
5	0.825	–0.072	0.997	0.06	0.966	0.159	0.472	0.300	0.027
Mean	0.787	–0.039	0.997	0.061	0.968	0.161	0.317	0.467	0.065
SD	0.041	0.036	0.001	0.002	0.003	0.017	0.193	0.275	0.051

### K-Folds cross-validation

We implemented a K-folds cross-validation procedure with 5 folds, such that we partitioned the data set into five independent training and test samples. We fitted a MixTree model with one to four number of classes in both partitions independently and compared their results according to some metrics.

First, we wanted to check if the estimated item parameters were similar in both training and test partitions. Therefore, we calculated the correlation and mean distance between item estimates obtained from training and test data sets. Second, we compared if the trait and ERS factor scores estimates and class membership probabilities are similar between when we use the estimates obtained from test model fit for the test data set and when we use the estimates obtained from the training model fit for the test data set. Finally, we also compared the overlap between class assignments between the two cases. We calculated the mean overlap and also Cohen’s $\kappa$ coefficient (Table 6).

The similarity between the item estimates obtained from the training and test partitions was the highest for the MixTree-2 model. The average correlation between the training and test partitions across five folds was 0.880 (SD=0.097), and the average distance between the partitions across five folds was 0.003 (SD=0.028). These values indicate high consistency, as compared to the MixTree-3 and MixTree-4 models, which demonstrated lower average correlations of 0.815 (SD=0.078) and 0.787 (SD=0.041), respectively, and slightly higher mean distances of -0.036 (SD=0.045) for MixTree-3 and -0.039 (SD=0.036) for MixTree-4.

For the trait factor, we observed almost a perfect correlation for the MixTree-2 model (Mean=0.999, SD=0.001). The MixTree-3 and MixTree-4 models also performed well, with mean correlations of 0.996 (SD=0.001) and 0.997 (SD=0.001), respectively, though they displayed slightly higher mean distances (MixTree-3: Mean=0.066, SD=0.006; MixTree-4: Mean=0.061, SD=0.002).

The deviation between the partitions becomes more salient with regard to the ERS score estimates. The MixTree-2 model demonstrated high consistency, with an average correlation of 0.997 (SD=0.001) and a mean absolute distance of 0.050 (SD=0.006), whereas the MixTree-3 and MixTree-4 models displayed correlations of 0.962 and 0.972, respectively, and greater mean distances of 0.17 (SD = 0.016) and 0.161 (SD = 0.017). These results suggest that the MixTree-2 model shows better consistency of the ERS factor scores between training and test partitions across 5 folds.

**Fig. 11:**
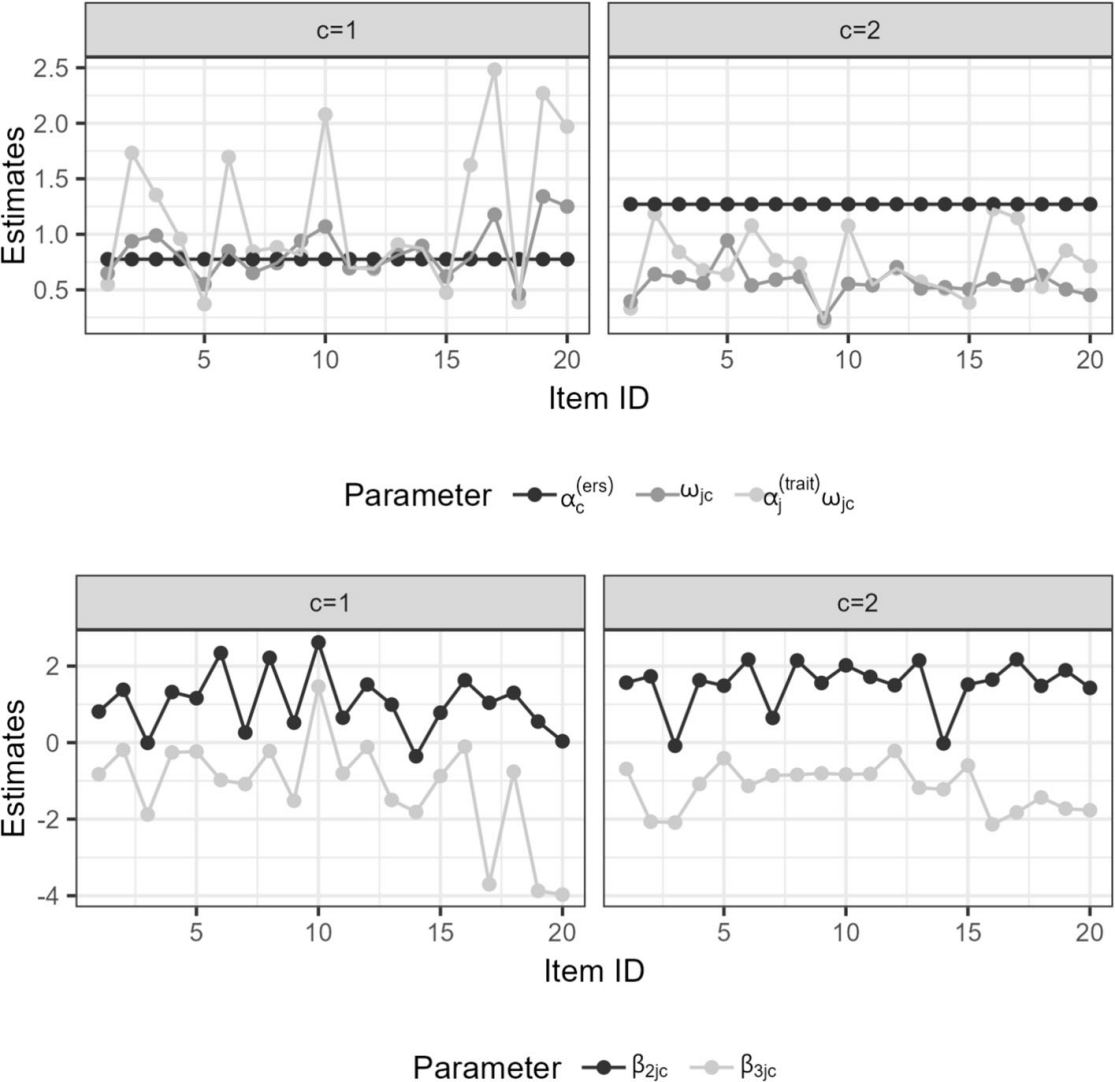
Parameter estimates of the second and third nodes obtained by MixTree-2 models. The *top panel* presents point estimates of the class- and item-specific proportionality constants $\omega _{jc}$ and class-specific ERS factor loadings $\alpha ^{(ers)}_{c}$. The *bottom panel* presents the node- item- and class-specific intercept parameters $\beta _{2jc}$ and $\beta _{3jc}$

Class assignment consistency was assessed using the mean overlap and Cohen’s $\kappa$. For MixTree-2, the average overlap (Hit) was 0.902 (SD=0.022), with a Cohen’s $\kappa$ of 0.800 (SD=0.041), indicating substantial agreement in class probabilities and assignments across folds. The MixTree-3 and MixTree-4 models had lower levels of overlap (0.297 and 0.467, respectively) and $\kappa$ values (0.268 and 0.065, respectively), indicating that class probabilities and class assignments were very divergent between when the test participants were classified with training estimates and when they were classified with the test estimates.

In summary, based on the similar information criteria and $R^{2}_{entropy}$ values across MixTree-2 to MixTree-4, and based on the superior performance of the MixTree-2 model in the K-Folds cross-validation, we concluded that the more parsimonious MixTree-2 model should be selected.

### Class-specific model parameters

In the top panel of Fig. 11, we present the contribution of the ERS factor (left-most; i.e., $\alpha ^{(ers)}_{c}$) and trait (right-most; i.e., $\alpha _{j}^{(trait)}\omega _{jc}$) in the decisions regarding extremity nodes. Moreover, in the middle, we present the class- and item-specific proportionality constants $\omega _{jc}$. In all figures, there is a clear difference between classes. First, we see in Class 1 that the total effect of trait captured with $\alpha _{j}^{(trait)}\omega _{jc}$ is greater the effect of ERS factor on category choices. In Class 2, we see the opposite pattern, where the ERS factor loading is greater than the effect of substantive trait on extreme category choices. All together, Class 1 is in line with a trait-dominated strategy, whereas Class 2 is more in line with a heuristic-dominated strategy. Second, we see that the proportionality constants were greater in Class 1 than in Class 2, implying that the trait involves more strongly in extreme category choices in Class 1 than in Class 2. Moreover, most estimates were smaller than 1 in both classes, implying that as previous research found (Alagöz & Meiser, 2024; Meiser et al., 2019), the trait involvement in the extremity decisions was weaker than the direction decision. Third, when we investigate the actual contribution of the trait in extremity decisions (i.e., the multiplication of the trait loadings at the first node and proportionality constants), we see a clearer divergence of classes from each other. That is, the term $\alpha _{j}^{(trait)}\omega _{jc}$ was much larger in Class 1 than in Class 2, implying that the trait played a stronger role in specific category choices in Class 1 than in Class 2.

As previously discussed, one advantage of the MixTree model is all person parameters have the same scale across classes through the class-invariant (co)variance matrix, allowing us to interpret the class-specific loadings very flexibly. For Class 1, we see that the ERS factor loading is mostly smaller than the trait factor loadings, meaning that the response processes in this class were more heavily affected by the trait of interest rather than the ERS factor. For Class 2, we see the opposite pattern. That is, the ERS factor loading is mostly larger than the trait factor loadings, meaning that the decision-making processes of respondents in this class were affected by ERS more heavily than the trait.

Since the scale identification involved setting latent means zero for all person parameters in all classes, we can also interpret the class-specific node intercepts comparatively. On the bottom panel of Fig. 11, we see no clear difference in the intercept terms $\beta _{2jc}$ and $\beta _{3jc}$ between classes. This finding means that both classes did not differ in the overall propensity of choosing category *2* over *1* and category *4* over *3* (see the tree structure in Fig. 1). A more substantive interpretation is that solely looking at the category frequencies or using mixture 1PL alike models (see Böckenholt & Meiser, 2017) might not be able to capture heterogeneity in response processes.

In conclusion, the two latent classes that we detected are associated with distinct response processes. Class 1 is associated with the stronger use of the trait in their response processes, whereas Class 2 is associated with heavier impact of the heuristic factor ERS. Referring back to the distinction that Krosnick (1991) made, Class 1 is more in line with an optimizing-dominated strategy and Class 2 with a satisficing-dominating strategy.

### Covariate effects

To have a better understanding of what factors might be affecting such differences in response processes, we further analyzed covariate effects on class membership as described in the third step of the estimation procedure. The data set contains two external variables that could be used in the analysis: gender (0=male, 1=female) and age. While keeping the original coding of the gender variable, we standardized the age variable to facilitate the estimation and interpretation. We fitted four regression models. One without any covariates, one with only the gender variable, one with only the age variable, and one with both variables. The information criteria (AIC, BIC) and likelihood-ratio tests suggested that both variables have significant effects (Table 7).

The results suggest that being female is associated with a higher probability of belonging to Class 2. In other words, females are more likely to follow a response process that is ERS-dominated. Regarding age, we found that older ages have a higher probability of belonging to Class 2, where the response process is ERS-dominated. Yet, these analyses were done on exploratory-basis and require a more structured investigation in the future, but it is not in the main goals of the present study.

**Table 7:** Covariate effects

Parameters	Class 2	SE [95% CI]
Intercept	-0.31	0.03 [-0.36, -0.26]
Gender	0.74	0.03 [0.67, 0.80]
Age	0.26	0.08 [0.09, 0.42]

Note: The reference group of the categorical gender variable is "male". SE: Standard Error. CI: Confidence Interval. Class 1 is specified as the reference group in the logistic regression analysis

In conclusion, we found two classes of remarkable sizes in the empirical data set: one with a stronger influence of the trait and one associated with a stronger influence of ERS on extreme category choices. For the overall propensity to choose extreme categories, the class-specific intercepts indicated no clear difference between classes. Finally, we found that females are more likely to follow a satisficing-dominated strategy and males are more likely to follow an optimizing-dominated strategy. Finally, the older the respondents are, the more likely it is that they follow a satisficing-dominated strategy.

## Conclusion

Under an ideal scenario, individuals would respond to questionnaire items based solely on their trait levels by, for example, following the four-step cognitive response process (comprehension, retrieval, integration, and mapping), referred to as optimizing strategy (Tourangeau et al., 2000). Each of these steps demands a considerable amount of cognitive effort. Therefore, when faced with the challenges such as lack of motivation, fatigue, comprehension issues, or unfamiliarity with construct/response scale, respondents may resort to heuristic processes to reduce their efforts, referred to as satisficing strategy (Krosnick, 1991). In such cases, the trait is no longer the sole determinant of the response, but additional factors like RS, or in most extreme cases effortless responding, come into play. Neglecting these heuristic response processes and exclusively modeling the trait can have significant effects on the measurement validity and subsequent inferences about the construct.

Several psychometric models were proposed to detect and correct for RS effects, such as IRTrees. However, these models assume that a single measurement model including RS factors hold for all respondents. There are two implications of this assumption. First, all respondents adopt a satisficing strategy. Second, the degree of satisficing is the same for all respondents through modeling a single set of traits and RS factor loadings. Both of these implications are ungrounded as several studies using discrete mixture models found evidence that there are subpopulations of optimizers, who use only the trait, and satisficers, who additionally employ RS in their response strategies (Alagöz & Meiser, 2024; Kim & Bolt, 2021; Tijmstra et al., 2018).

It is rather constraining to consider satisficing and optimizing as binary outcomes. The mixture models mentioned above use a confirmatory approach with predefined latent classes, where there is only the trait determining the responses to capture optimizers, and where there is additional RS factors to capture satisficers. However, the factors interrupting the four-step process may not be the same for all respondents and may not have the same severity. That is, while some respondents are extremely tired and skip several steps, some others can be only slightly tired to go through all steps but with less effort, and some others may be fully engaged and execute all steps with full attention. Thus, a more flexible mixture approach is needed that accommodates gradual degrees of satisficing and optimizing, respectively.

The proposed MixTree model addresses all the problems mentioned above. First, by having the mixture components, we do not put the strict assumption that all respondents follow a single response strategy. Moreover, in contrast to previous mixture models, the MixTree does not fix any factor loadings at zero in any class. Thereby, we do not enforce that respondents follow either a satisficing or an optimizing strategy, but respondents can put different weights to the trait and ERS factor that are not pre-determined. Respondents can thus also follow a balanced strategy, and any combination of the response strategies can be detected. The MixTree model also investigates the external variables that may predict class memberships, allowing further insights on the differential use of trait and ERS factors.

The simulation study showed great performance of the MixTree model in recovering class sizes, class memberships, and covariate effects in different class sizes, class separation, trait-ERS correlation, test lengths conditions, except for the shortest test length (J=10) and lowest class separation $(R^{2}_{entropy})$. Furthermore, the MixTree can recover item and person parameters with minimal error. We also found that under realistic conditions, the AIC proved itself as a useful tool for determining the number of classes, especially in cases with smaller sample sizes. Yet, such decisions should be aided with the $R^{2}_{entropy}$ statistic, substantive interpretation of class definitions, and an absolute fit investigation with, for example, K-folds cross-validation methods.

When one does not account for existing heterogeneity by fitting one class or fewer than the true number of classes, class-specific item parameters and ERS factor scores were recovered with bias. Meanwhile, the trait scores showed minimal error as the class-invariant first node provides sufficient information even with fewer than the true number of classes. Yet, the measure of precision for the person parameters was somewhat off when heterogeneity was not accounted for adequately. These findings show that under-enumeration of classes may result in biased estimates, whereas over-enumeration is mostly unproblematic for the estimation part, but it may make the interpretation of classes more difficult.

Although the single-class IRTree model can yield accurate trait estimates in heterogeneous populations, primarily because the first node is shared across all classes and captures the bulk of trait-related variance, the core contribution of the MixTree model lies beyond trait estimation. Specifically, MixTree allows us to empirically model and test whether individuals differ in how they arrive at their responses. Traditional IRTree models account for response styles but assume homogeneity in response strategies across individuals. In contrast, MixTree introduces latent classes that reflect qualitatively distinct response strategies (e.g., trait-based vs. heuristic-based), drawing on theoretical insights from the satisficing literature. Thus, the primary value of MixTree is not simply in improving trait scores, but in uncovering meaningful individual differences in response processes that standard models cannot capture.

Following the simulation study, we illustrated with empirical data that respondents indeed follow different response strategies. We found two sub-populations of respondents, one associated with stronger influence of the trait on extreme decisions than the ERS factor and one with stronger influence of the ERS factor than the substantive trait. The difference between the classes was noticeable as the average trait loadings across items (1.18) were higher than the ERS loading (0.75) in the former class, whereas the ERS factor had a stronger impact (1.25) than the substantive trait (0.73) in the latter class. The first class, by having stronger trait loadings than the ERS loading, can be considered more in the optimizing direction, whereas the second class, by having a stronger ERS loading than the trait loadings, is towards the satisficing direction. The cross-validation held via K-folds with five folds revealed a great overlap of item, person, and class parameter estimates across five different pairs of training and test subsets, whereas for MixTree models with higher number of classes, the training and test data metrics showed mismatch that points at potential overfitting in these cases.

We also illustrated how class predictors can be included in the model. We found that, in this specific empirical illustration, being female and having an older age increases the probability of following an ERS-dominated strategy. When available, different types of predictors, especially process data (e.g., response times) can be used for validating or gaining more insights about the classes (e.g., Khorramdel et al., 219).

The present approach extends previous models such that it does not enforce specific response strategies through fixing some factor loadings to zero or by limiting the number of classes beforehand. Moreover, previous models predefined two latent classes, representing the optimizers and the satisficers (Alagöz & Meiser, 2024; Kim & Bolt, 2021; Tijmstra et al., 2018). The MixTree, on the other hand, does not predefine any response strategy or force respondents to belong to any of the pre-fixed number of classes, but it is capable of capturing more gradual differences in the response strategies between latent classes, not necessarily as optimizers or satisficers. As is shown in our illustration, respondents can still make use of the trait in the satisficing and ERS in the optimizing strategy to a substantial degree.

Naturally, our work has some limitations that future research should address. First, we assumed that all respondents comprehend the items to the same extent by having a class-invariant response direction node. However, the MixTree model can be adjusted to investigate many scenarios. In extreme cases, it may be the case that some respondents skip the comprehension step, which may result in the (dis)acquiescence RS (i.e., tendency to choose (dis)agreement categories). In such cases, one can further incorporate the acquiescence RS factor in the response direction node with class-specific weights of the trait and RS. Alternatively, the MixTree can be used to capture effortless responding by keeping the first node unidimensional with the substantive trait but with class-specific factor loadings, which would then capture overall attentiveness of the respondents. For instance, weak trait loadings would imply that the trait does not play a significant role in the entire response process, and the respondents rather engage in *random* or *non-effortfull* responding (Ulitzsch et al., 2024, 2022). Additionally, response biases such as socially desirable responding is also found to be qualitatively different between subpopulations (Seitz et al., in press), which could be of interest to incorporate in the MixTree approach. Related to the discussion point of good recovery performance of trait scores with the single-class IRTree model in heterogeneous populations, such model extensions would further highlight the need for accounting for heterogeneity to debias the trait scores and loadings.

Second, the class separation conditions were generated by using rather arbitrary values of $R^{2}_{entropy}$ values, since our study is the first to our knowledge that investigates such an entropy-reduction measure in the mixture modeling of response process heterogeneity. It is possible that our choices for the low separation condition was way lower than the reality. However, with investigating three levels of separation, we covered a wide range and those who apply the MixTree may take these values as a reference.

Third, to keep the MixTree as parsimonious as possible, we modeled the ERS factor loading $\alpha ^{(ers)}_{c}$ as item-invariant. This decision was not arbitrary and is based on the theories regarding the RS. More specifically, RS are by definition independent of the item-content, and they should not be item-specific. However, some external factors, such as item length or wording can make some items more prone to heuristic strategies. However, one can easily extend the model MixTree to contain a class- and item-specific $\alpha ^{(ers)}_{jc}$, but this specific issue was not our primary goal and therefore future studies can test this specific assumption.

Fourth, we only considered continuous and uncorrelated covariates. However, as the measurement and structural model are estimated separately via a three-step ML approach (Vermunt, 2010), the results of the previous studies investigating specific settings of the structural model on the recovery of covariate effects would apply to the MixTree model as well (Alagöz & Vermunt, 2022; Bakk et al., 2013, 2014; Bakk & Kuha, 2021).

Fifth, the presented version of the MixTree can handle only four-point rating scale items, and thus the ERS factor. Yet, it can easily be extended to six-point rating scale items and to model also MRS. For five-point rating scale items, we refer readers to Alagöz and Meiser (2024), where they demonstrate how to include trait, ERS, and MRS effects with odd numbers of categories.

And finally, not necessarily a limitation, the simulation results suggested that the MixTree needs longer tests than ten items, larger sample size than 1000, and a higher class separation than $R^{2}_{entropy}=0.20$ to show satisfactory performance.

In conclusion, we introduced the MixTree approach for disentangling different response mechanisms in rating scale data. With an extensive simulation study, we depicted the wide range of capabilities of the MixTree model in detecting heterogeneity regarding response strategies, such as accurate classification of respondents, recovery of class-specific item and person parameters with minimal biases, and accurately recovering covariate effects on class membership. As illustrated with an empirical data set, the MixTree can provide a deeper insight on how individuals differ in the way they respond compared to other models in the literature, and can be of great use for psychometricians and applied researchers in expanding our knowledge on behavioral aspects of responding in questionnaires.

## Data Availability

The empirical data set used for illustration is available at https://openpsychometrics.org/_rawdata/. Generated data sets concerning the simulation study are available on OSF: https://osf.io/zs3jq/.

## Footnotes

1 Depending on the number of response categories used, the structure and the types of RS we can account for changes. See Alagöz and Meiser (2024) for five-point rating scale items and Debelak et al. (2024) for six-point rating scale items.

2 The chosen parameter values for the weights of $\theta$ and $\eta$ compensate each other in each class (i.e., add up to one). Such compensation between the trait and ERS loadings is a simplifying assumption only for the hypothetical example and not a requirement in the MixTree approach. It can be the case that class-specific parameters are proportional between classes, for example, $\alpha ^{(ers)}_{c}=\{0.6, 0.3\}$ and $\omega _{jc}=\{0.9, 0.45\}$. Then, both classes follow a similar strategy regarding the relative weighting of $\theta$ and $\eta$. That is, within each class, the trait has a higher weight, thus influences the response strategy more than the ERS. Yet, in the second class, neither the trait nor the ERS explains the variance in extremity decisions considerably. Then, one can infer that the response strategy is either dominated by factors that are not accounted for in the model, or subjects respond more in line with effortless responding so that the trait or ERS cannot explain the variance (see Ulitzsch et al., 2024).

3 Throughout the text, a *+* subscript is used to indicate the entire column or row of a matrix

4 We also found no convergence problems in neither $\sigma (\theta , \eta )=0$ nor $\sigma (\theta , \eta )=0.3$ conditions, and the mean duration of estimation was almost the same. With a higher level of correlation, such problems may occur, but our choice for the parameter value was based on empirical findings (Alagöz & Meiser, 2024; Böckenholt & Meiser, 2017; Merhof & Meiser, 2024; Merhof et al., 2024). Furthermore, the information on $\theta$ that the class-invariant first node brings in the estimation may have facilitated the parameter recovery regardless of the correlation level.

5 https://openpsychometrics.org/_rawdata/

## Code Availability

The codes used to estimate the models are available on OSF: https://osf.io/zs3jq/.
